# Identification and characterization of LysM effectors in *Penicillium expansum*

**DOI:** 10.1371/journal.pone.0186023

**Published:** 2017-10-30

**Authors:** Elena Levin, Ana Rosa Ballester, Ginat Raphael, Oleg Feigenberg, Yongsheng Liu, John Norelli, Luis Gonzalez-Candelas, Jing Ma, Christopher Dardick, Michael Wisniewski, Samir Droby

**Affiliations:** 1 Department of Postharvest Science, ARO, the Volcani Center, Bet Dagan, Israel; 2 Instituto de Agroquímica y Tecnología de Alimentos (IATA-CSIC), Avda. Agustin Escardino, Paterna, Valencia, Spain; 3 School of Biotechnology and Food Engineering, Hefei University of Technology, Hefei, China; 4 Appalachian Fruit Research Station, USDA-ARS, Kearneysville, WV, United States of America; Universita degli Studi di Pisa, ITALY

## Abstract

*P*. *expansum* is regarded as one of the most important postharvest rots of apple fruit and is also of great concern to fruit processing industries. Elucidating the pathogenicity mechanism of this pathogen is of utmost importance for the development of effective and safe management strategies. Although, many studies on modification of the host environment by the pathogen were done, its interactions with fruit during the early stages of infection and the virulence factors that mediate pathogenicity have not been fully defined. Effectors carrying LysM domain have been identified in numerous pathogenic fungi and their role in the first stages of infection has been established. In this study, we identified 18 LysM genes in the *P*. *expansum* genome. Amino acid sequence analysis indicated that *P*. *expansum* LysM proteins belong to a clade of fungal-specific LysM. Eleven of the discovered LysM genes were found to have secretory pathway signal peptide, among them, 4 (*PeLysM1 PeLysM2*, *PeLysM3* and *PeLysM4*) were found to be highly expressed during the infection and development of decay of apple fruit. Effect of targeted deletion of the four putative PeLysM effectors on the growth and pathogenicity was studied. Possible interactions of PeLysM with host proteins was investigated using the yeast-two-hybrid system.

## Introduction

Blue mold of pome fruit, caused by *Penicillium expansum*, is regarded as one of the most important postharvest rots of apple fruit [1, 2]. Financial losses resulting from postharvest decay of apple, including blue mold, can exceed 4.5 million dollars per year in the United States alone [3]. *P*. *expansum* is also of great concern to fruit processing industries (juicing, baby food, ready to eat salads) due to its production of patulin, a highly toxic mycotoxin which can contaminate infected produce and processed products [4, 5]. Clearly, postharvest losses due to *P*. *expansum* represent a significant issue.

*P*. *expansum* infects fruit hosts through surface wounds where nutrients stimulate spore germination, which is then followed by penetration and colonization of fruit tissue. Modification of or adaptation to the host environment by the pathogen is considered to be a major determinant for successful colonization of host tissues [6, 7, 8]. Regulation of pH in fruits plays an important role in postharvest disease because it directly affects the germination of spore [9] and influences the virulence of pathogens [7, 10]. The role of pH and pectolytic enzymes in the *P*. *expansum/*host interaction has been intensely studied [10, 11, 12]. The virulence factors that mediate pathogenicity in postharvest-decay fungi, however, have not been fully defined, including if and how the pathogen suppresses fruit defense mechanisms, especially in the *Penicillium*-apple interaction.

Secreted effector proteins enable plant pathogenic fungi to manipulate host defenses for successful infection. Typically, these effectors are relatively small proteins that are secreted either through classical systems (requiring a signal peptide and transit through the endoplasmic reticulum and Golgi) or, alternatively, in some species, may be secreted through a non-classical pathway [13, 14, 15]. Their putative and, in some cases, proven function is to enable colonization by the pathogen via interfering with PAMP-triggered immunity (PTI) that might otherwise hinder pathogen colonization. Effector-triggered immunity (ETI), the second branch of plant defense, frequently conforms to a gene-for gene model [16] whereby immunity is the result of a single gene/loci in the pathogen that encodes a protein that interacts with a protein in the plant, the latter of which is encoded by a single dominant gene/loci. An inverse of the gene-for-gene model has also been described that involves an interaction between individual effector genes from the pathogen and single dominant susceptibility loci in the plant [17]. In the latter scenario, the interaction promotes plant infection through the activation of plant cell death signaling pathways often resembling those triggered during effector-triggered immunity [18]. These mechanisms, described as effector-triggered susceptibility [17], have been shown to favor plant infection by various necrotrophic fungi, and in particular two wheat (*Triticum aestivum*)-infecting fungi, *Stagonospora nodorum* and *Pyrenophora tritici* [19], which are the causal agents of leaf blotch and tan spot diseases, respectively.

These observations raise an intriguing question regarding the molecular interaction between necrotrophic fungal pathogens and their host plants. It is assumed that necrotrophic pathogens do not comply with the gene-for gene model. Several necrotrophic fungal pathogens, however, have been reported to produce proteins, which might function as effectors that induce necrosis in plants via their interaction with compatible host proteins associated with host defense [17]. Among the most studied fungal effectors are proteins possessing Lysin motifs (LysM) that function in prokaryotes and plants in carbohydrate-binding protein modules [20, 21]. In *Cladosporium fulvum*, LysM effectors were found to act as virulence factors during colonization [22]. It has been suggested that LysM-containing proteins are widespread among fungi of diverse taxa and lifestyles [20]. Since LysMs can bind chitin oligosaccharides, it has been proposed that many fungal LysM proteins might be involved in sequestering chitin oligosaccharides in order to prevent elicitation of host immune responses or inhibit attraction of mycoparasites [23], and/or the protection of fungal hyphae against chitinases secreted by competitors [20]. In contrast, bacterial LysM-containing proteins are mostly peptidoglycan hydrolases [24].

Genetic homologues of lysine motif (LysM) effectors have been identified in many fungal species [13]. Effectors with LysM were reported to play a major role in the virulence of several plant pathogens [13, 15, 22, 25, 26, 27, 28]. Ecp6, a chitin-binding protein from *Cladosporium fulvum*, appears to prevent the activation of PTI by scavenging chitin oligosaccharides that act as pathogen-associated molecular patterns (PAMPs). Mg3LysM, a protein from *Mycosphaerella graminicola*, blocks the elicitation of chitin-induced plant defenses, and was shown to protect fungal hyphae from plant-derived hydrolytic enzymes [25]. Moreover, Mg3LysM mutant strains were severely impaired in leaf colonization, did not trigger lesion formation, and were unable to undergo asexual sporulation [25]. Slp1 is required by Magnaporthe oryzae for full virulence and exerts a significant effect on tissue invasion and disease lesion expansion. It accumulates at the interface between the fungal cell wall and the rice plasma membrane, can bind to chitin, and is able to suppress chitin-induced plant immune responses, including the generation of reactive oxygen species and plant defense-related gene expression [26]. In *Colletotrichum higginsianum*, two LysM effectors ChELP1 and ChELP2 were found to suppress the chitin-triggered activation of two immune-related plant mitogen-activated protein kinases in the host Arabidopsis and are shown to be essential for fungal virulence and appressorium-mediated penetration of both Arabidopsis epidermal cells and cellophane membranes in vitro [27]. However, in *Verticillium dahlia*, only lineage-specific LysM effector of strain VdLs17 was reported to contribute to virulence through suppression chitin-induced immune responses and protection fungal hyphae against hydrolysis by plant hydrolytic enzymes. Other three core LysM effectors that are conserved in a collection of *V*. *dahliae* strains, not expressed *in planta*, are not contributing to virulence [28]. Fungal LysM proteins are also involved in the self-regulation of fungal growth and development. For example, TAL6 from the plant-beneficial fungus *Trichoderma atroviride* was shown to have a strong inhibitory effect on spore germination. TAL6 specifically inhibits the germination of *Trichoderma* spp., but not other fungi. Thus, this protein is involved in self-signaling processes during fungal growth [29]. In a comprehensive review, Sperschnieider et al. point out that while molecular studies have identified over 60 fungal effectors from different species, this figure likely represents only a small fraction of the total of fungal effector proteins [30].

The focus in the present study was to characterize the LysM effector family in *P*. *expansum* and to investigate their potential role in its pathogenicity on apple fruit. An annotated genome sequence of *P*. *expansum* was used to identify putative LysM effectors based on similarity to known sequences found in public databases. Based on transcriptomic data obtained during the infection and development of *P*. *expansum* decay on apple, four secreted and expressed LysM effectors were characterized and functionally analyzed.

## Materials and methods

### Bioinformatic analysis of LysM sequences

The prediction of the LysM domains in *P*. *expansum* proteins and the identification of their location within a protein were done using InterPro v.60.0 [31] and PROSITE Release 20.123 of 09-Feb-2016 [32]. Prediction of secretory pathway signal peptide presence was done by TargetP 1.1 [33]. LysM alignment of all the predicted LysM carrying proteins found in *P*. *expansum* was done using T-Coffee (http://www.ebi.ac.uk/Tools/msa/tcoffee/) [34, 35, 36] and a consensus pattern was constructed using GLAM2 version 4.11.1(http://meme-suite.org/tools/glam2) [37]. Visualization of the alignment was done using Jalview [38].

The alignment of PeLysM1, PeLysM2, PeLysM3, and PeLysM4 LysM domains to the LysM domains of effectors reported in the literature was done by MEGA6 software using ClustalW algorithm. The phylogenetic tree was constructed using best neighbor algorithm.

### Plant material and fungal cultures

Apple fruits (cv. Golden Delicious) were obtained from fruit summits packing house Agricultural Cooperative Association Ltd. (Kibutz Iftah 1384000, Israel) and used shortly after harvest or stored at 0°C until use. Prior to inoculation, fruit were disinfected by drenching in a water solution of 0.05% sodium hypochlorite for 2 min and then washed thoroughly with tap water.

A culture of *P*. *expansum* (strain PE100/PEX2) was cultivated on potato dextrose agar (PDA) or in liquid culture in potato dextrose broth (PDB) at 25°C. Spore suspensions were prepared by harvesting the spores from 2 to 3-weeks-old PDA cultures with a sterile bacteriological loop, and suspending them in sterile distilled water. To remove residual mycelium, the suspension was filtered through four layers of sterile cheesecloth and spores were washed twice with sterile distilled water by centrifugation at 10,000 rpm for 5 min and resuspension in sterile distilled water. Spore concentration was determined using hemocytometer and adjusted as needed.

### Fruit inoculation and disease assessment

Apple fruits were wounded at four to six different sites around the stem end to a depth of 4 mm using a 1.25-mm diameter needle. For inoculation, 10 μl of a spore suspension of *P*. *expansum* (10^6^ spores ml^-1^) was pipetted into each wound. For each treatment, 8–12 fruits were used and each experiment was repeated three times. Inoculated fruit was placed in plastic trays lined with filter paper soaked with sterile water (150 ml per tray) and incubated in darkness at 25°C. Percentage of infection and rot diameter were determined starting from the 3rd day after inoculation.

### RNA extraction from *P*. *expansum*-infected apple tissue and cDNA synthesis

Total RNA was extracted from *P*. *expansum*-infected apple fruit tissue at different times after inoculation following the protocol described by Chang *et al*. (1993) [39]. Decayed tissues from the site of inoculation were removed from apples, frozen immediately in liquid nitrogen and then freeze-dried. For each sample, 1gr of lyophilized tissue was ground in liquid nitrogen using a mortar and pestle. The ground powder was suspended in 10 ml of extraction buffer (2% C-TAB, 2% polyvinylpyrrolidone (PVP), 0.1M Tris-HCl (pH8), 25 mM EDTA, 2M NaCl, 2% β-mercaptoethanol) preheated to 65°C. After vortexing for 2 min, the samples were incubated at 60°C for 15 min with occasional mixing. After incubation, an equal volume of Chloroform:Isoamyl alcohol (Chl:Iaa) (24:1 v/v) was added to each sample, vortexed for 2 min and centrifuged at 12,000g for 20 min at 4°C. The upper phase was transferred to a clean tube and the RNA was re-extracted with an equal volume of Chl:Iaa. The supernatant was then transferred to a clean 50 ml tube, mixed with 1/3 volume of 7.5M LiCl and incubated overnight at 4°C. RNA was precipitated by centrifugation at 12,000g for 50 min at 4°C. The pellets were washed with 5 ml 75% EtOH, air dried, and dissolved in 500 μl of preheated (65°C) SSTE buffer (0.5% SDS, 1 M NaCl, 10 mM Tris-HCl (pH 8), 1mM EDTA). The RNA was re-extracted again by adding an equal volume of Chl:Iaa, vortexed for 2 min and subjected to centrifugation at 14000g for 10 min at 4°C. The upper phase was transferred to a clean microcentrifuge tube. RNA was precipitated by the addition of 2 volumes of 100% ethanol, incubation at -80°C for 30 min, and centrifugation at 14,000g for 20 min at 4°C. The pellets were washed with 1 ml 75% ethanol, air dried, and dissolved in 50 μl of nuclease-free water. The quality and concentration of total RNA was analyzed by gel electrophoresis and the use of a ND-1000 spectrophotometer (NanoDrop, Wilmington, DE, USA). First-strand cDNA was synthesized from 1 μg of total RNA that had been pretreated with 0.25 units of recombinant DNase I (Takara Bio Inc., Shiga, Japan), using the Verso cDNA Kit (Thermo Fisher Scientific, Epson, UK) following manufacturer's guidelines.

### PCR and reverse transcription–quantitative PCR (RT-qPCR)

PCR reactions were done with standard thermocyler (Senso Quest, Göttingen, Germany). Each PCR reaction contained 1U DreamTaq DNA polymerase (Thermo Scientific), DreamTaq buffer, 0.2 mM dNTPs, 0.4 μM of each primer as detailed below and 1 ng–1μg template DNA. Two sets of cycling conditions were used: 1) 5 min at 95°C, followed by 36 cycles of 45sec at 94°C, 45sec at 53°C, 45sec at 72°C; and 10 min at 72°C, and 2) or 5 min at 94°C, followed by 40 cycles of 30 sec at 94°C, 15 sec at 54–58°C, 1–3 min at 72°C; and 10 min at 72°C. Primer sequences utilized in the PCR reactions are listed in S1 Table

RT-qPCR was performed with a Rotor-Gene 3000 system (Corbett Research, Sydney, Australia). PCR amplification was performed with 2.5 μl of cDNA template in 10 μl of a reaction mixture containing 5 μl of ABsolute™ Blue qPCR SYBR® Green ROX Mix (Thermo Scientific, ABgene legacy), and 300 nM primers as detailed below (S1 Table).

Results were analyzed using Rotor-Gene 6 software. For the determination of gene expression as function of time, 28S rRNA, 37S ribosomal protein s24, and Histone H3 served as endogenous controls. The sample collected at 0 hours post inoculation (hpi) were used to calibrate the expression levels. A mixture of cDNAs from all the samples was used in the analysis of all the treatments as a template to generate calibration curves designed for each pair of primers. This was done to verify that a single PCR product had been generated. Relative quantification was calculated using the mathematical model of Pfaffl (2001) in which the relative expression ratio of a target gene is calculated based on the efficiency and the cross point deviation of an unknown sample versus a control [40], and expressed in comparison to geometrical mean of the three reference genes indicated above Each experiment was performed in triplicate, and three different biological experiments were conducted.

### Construction and analysis of knockout mutant

Knockout mutants were constructed as described previously by Ballester et al. (2015) [41]. Flanking regions of the 5′ upstream (promoter) and 3′ downstream (terminator) of *LysM1*, *LysM2*, *LysM*3 and *LysM*4 genes, were PCR amplified from genomic DNA of *P*. *expansum* (Pe100/PEX2), using specific primer pairs (S1 Table). The cycling protocol consisted of 4 min at 94°C for pre-incubation, then 40 cycles of 30 s at 94°C for denaturation, 15 s at 56°C for annealing, and 2 min at 72°C for extension, and a final elongation step of 10 min at 72°C. DNA fragments corresponding to the promoter and terminator regions, and the pRFHU2 vector [42] were digested with *PacI* and *NtBbvCI* (New England Biolabs), mixed together and then treated with USER enzyme (New England Biolabs) to obtain the plasmids pRFHU2-LysM1, pRFHU2-LysM2, pRFHU2-LysM3 and pRFHU2-LysM4. An aliquot of the mixture was used directly for chemical transformation of *Escherichia coli* JM109 cells (Promega) without prior ligation. Kanamycin-resistant transformants were screened by PCR for the presence of promoter and terminator with primer pairs RF-1/RF-6 and RF-2/RF-5, respectively (S1 Table). Proper insertion was confirmed by DNA sequencing. The selected plasmids were introduced into chemically competent *Agrobacterium tumefaciens* AGL-1 cells, which were subsequently used to transform *P*. *expansum* PEX2, as described previously [43]. Analysis of transformants for gene disruption events was done by PCR. The insertion of the selection marker was checked with primer pair 3/4 (S1 Table). Integration of the T-DNA by homologous recombination was examined using primer pairs 5/6 and 7/8 for *PeLysM1*, 11/6 and 7/12 for *PeLysM2*, 15/6,7/16 for *PeLysM3* and 19/6 and 20/7 for *PeLysM4* (S1 Table). Further verification of deletion of the target gene was done with primer pairs 1/2, 9/10, 13/14, and 17/18 for *PeLysM1*, *PeLysM2*, *PeLysM3*, and *PeLysM4*, respectively (S1 Table). Real-time genomic PCR analysis was carried out in order to determine the number of T-DNA copies that had been integrated into the genome of the transformants, following the protocol described by Crespo-Sempere et al. (2013) [44], using PEX2 as the control. Primer pairs, 23/24, 25/26, 27/28, and 29/30 were designed within the T-DNA in the promoter regions of *PeLysM1*, *PeLysM2*, *PeLysM3*, and *PeLysM4*, respectively, close to the selection marker. The *P*. *expansum* β-tubulin gene (AY674401) [45] was chosen as reference gene using the primer pair 21/22. The expression of *PeLysM1*, *PeLysM2*, *PeLysM3*, and *PeLysM4* in the null mutants, ectopic mutants and wild type was determined using RT-PCR or RT-qPCR as previously described.

### Radial growth, sporulation, and spore-germination assays

For radial growth assay, 5-μl droplet of spore suspension of 10^6^ spore/ml was placed in the center of PDA plates, incubated at 25°C and culture diameter was recorded daily. For quantification of spore production, discs of 1.5 cm diameter was cut from the center of 11 days-old plaits and placed in a sterile tube containing 5 ml distilled water. Spores were released from the mycelium by vortexing the tube for 30 sec., and spore concentrations were counted by a hemocytometer. To examine the percent of spore-germination and germ-tube length, 15-μl droplet of 10^5^ spore/ml were inoculated on water-agar plates (1.5% agar). After incubation at 25°C for 18 hr the plates were observed under a microscope and image analysis and germ-tube length measurements were done using NIS-Elements BR Microscope Imaging Software (Nikon Instruments Inc., USA).

### Yeast two hybrid

Y187 and Y2H Gold yeast strains were used in the yeast two hybrid experiments. Yeast were grown on YPDA or selective minimal medium. Yeast transformation was conducted using the Yeastmaker yeast transformation system 2 (Clontech Laboratories, Inc. Mountain View, CA 94043 USA. Cat.N0. 630439).

For the construction of the GAL4 Activation Domain plasmid (Gal4-AD prey cDNA library), total RNA was isolated from ‘Royal Gala’ apple fruit collected at three different stages of development: early (40 mm in diameter), mid-season, and mature (harvest stage). Fruit were either left unwounded, wounded, or wounded-inoculated with *Penicillium expansum*. RNA was extracted from the fruit tissues as described by Ballester et al. (2015) [41] and pooled prior to generating cDNA using a SMART cDNA synthesis kit (ClonTech CA USA. Cat. No. 630490) according to the manufacturer’s instructions. The library was packaged into the pGADT7-Rec vector which was then used to transform yeast strain Y187.

Construction of the GAL4 DNA binding domain plasmid (Gal4 Bait DNA-BD): *PeLysM1* ORF was amplified from cDNA using LysM1_complete_F and LysM1_complete_R primers and cloned into pGEM_T Easy vector (Promega USA. Cat. No. A1360) to construct pGEM+LysM1. LysM (used as a bait) was amplified from pGEM+LysM1 by PCR using a 24 bp primer with homology to the bait and a 16 bp primer with homology to the linear ends of pGBKT7. The LysM PCR fragment and linearized pGBKT7 vector were mixed together and “fused” using an in-fusion enzyme (ClonTech CA USA. Cat. No. 638909). The bait plasmid was then transformed into the Y2H Gold yeast strain.

Yeast Two-hybrid Assay: Yeast two-hybrid screening was performed using the Matchmaker Gold Yeast Two-Hybrid System (ClonTech CA USA. Cat. No.630489). The resulting candidate yeasts cell could potentially possess one or more prey plasmids that do not express an interacting protein (false positives). Therefore, to increase the chance of rescuing positive prey plasmids, the candidate yeast cells were re-streaked several (3–4) times on SD/-Leu-Trp (DDO)/X selective medium. Each time a single blue colony, indicating a positive interaction (blue), was picked for re-streaking. All positive interactions (clones) were confirmed by patching on high stringency screening medium (SD/-Ade-His-Leu-Trp (QDO)/X/A). The sequence of the inserted prey plasmids was obtained by PCR amplification with primers specified by the manufacturer. The insertions were then sequenced by Macrogen, Inc. (Bethesda, MD, USA). In order to further confirm that the interactions were genuine, prey plasmids from *E*. *coli* were transformed into the Y2H yeast strain containing the bait plasmid and grown on highly selective medium QDO/X/A.

### Statistical analysis

Statistical analysis of strain pathogenicity and radial growth was based on nested one-way ANOVA followed by Tukey’s honest significant difference (HSD) test on a JMP 13.0 platform (SAS Institute, Cary, NC).

## Results and discussion

### Identification of LysM effector homologs in the genome sequence of *P*. *expansum*

Although the majority of known fungal effectors are unique and share little similarity with known proteins [20], effectors carrying LysM domain have been identified in numerous pathogenic fungi and their role in the first stages of infection has been established [22, 25, 26, 27, 28]. Analysis of a database of 650 000 predicted proteins from 70 fungal species encompassing pathogenic and non-pathogenic fungi revealed that the number of LysM proteins in a genome can vary greatly from 0 up to 172 proteins [20]. We searched the *P*. *expansum* PEX2 genome using IPR018392 to identify 18 genes possessing a LysM domain. Eleven of the LysM genes have a signal peptide and are thus predicted to be secreted (Table 1). Two of the genes (PEX2_040510, PEX2_091440) contain four LysM domains, two of the genes (PEX2_101830, PEX2_060980) contain three domains, five of the genes (PEX2_025780, PEX2_030940, PEX2_003000, PEX2_060940, PEX2_091460) contain two domains, and the other eight genes, contain one LysM domain (Tables 1 and 2). All of the genes encode cysteine-rich proteins with 8–46 cysteines each. The size of the predicted gene products varied between 231 to 1500 amino acids. Among the 18 LysM genes, there are two (PEX2_101830, PEX2_00300) with a pectin-lyase fold (IPR012334), and one gene (PEX2_020570) with a lysozyme-like domain (IPR023346). There are also three genes (PEX2_025780, PEX2_063960, PEX2_091460) that contain a chitinase domain. Two of them (PEX2_025780, PEX2_ 091460) also have an Ecp2 effector domain (IPR029226) at their C-terminus. Although these proteins, have LysM that are not considered as effectors and could potentially belong to catalytic proteins modules involved in the degradation of exogenous chitin as well as the remodeling and recycling of chitin within the fungal cell wall [21].

**Table 1:** Genes containing LysM with predicted signal peptide found in *P*.*expansum* genome.

Name	Predicted product size (aa)	# of LysM domains	# of Cys residues	Blast2Go annotation	Additional domains
PEX2_025780	1500	2	46	Peptidoglycan-binding Lysin subgroup	Chitin-binding type 1 (IPR001002), Glycoside hydrolase superfamily (IPR017853),Glycoside hydrolase, catalytic domain (IPR013781),Glycoside hydrolase family 18, catalytic domain (IPR001223),Chitinase II (IPR011583),chitinase insertion domain (IPR029070), Ecp2 effector domain (IPR029226)
PEX2_008300	276	1	13	hypothetical protein	
PEX2_101660	384	1	20	Peptidoglycan-binding Lysin subgroup	Chitin-binding type 1 (IPR001002)
PEX2_060980	241	3	11	Peptidoglycan-binding Lysin subgroup	
PEX2_030940	543	2	25	Peptidoglycan-binding Lysin subgroup	Chitin-binding type 1 (IPR001002)
PEX2_033310	231	1	8	Peptidoglycan-binding Lysin subgroup	
PEX2_020570	422	1	11	Peptidoglycan-binding Lysin subgroup	Lysozyme-like domain (IPR023346)
PEX2_091440	344	4	16	Peptidoglycan-binding Lysin subgroup	
PEX2_095570	376	1	18	Peptidoglycan-binding Lysin subgroup	Chitin-binding type 1 (IPR001002)
PEX2_053590	316	1	16	Peptidoglycan-binding Lysin subgroup	Chitin-binding type 1 (IPR001002)
PEX2_064530	542	2	25	hypothetical protein	

**Table 2:** Genes containing LysM without signal peptide found in *P*.*expansum* genome.

Name	Predicted product size (aa)	# of LysM domains	# of Cys residues	Blast2Go annotation	Additional domains
PEX2_101830	1410	3	33	Peptidoglycan-binding Lysin subgroup	Pectin lyase fold/virulence factor (IPR011050),Pectin lyase fold (IPR012334)
PEX2_040510	580	4	28	Peptidoglycan-binding Lysin subgroup	
PEX2_080870	300	1	11	hypothetical protein	
PEX2_003000	1258	2	31	Pectin lyase fold/virulence factor	Pectin lyase fold/virulence factor (IPR011050), Pectin lyase fold (IPR012334)
PEX2_060940	769	2	29	Peptidoglycan-binding Lysin subgroup	Chitin-binding type 1 (IPR001002)
PEX2_063960	1116	1	30	Peptidoglycan-binding Lysin subgroup	Glycoside hydrolase superfamily (IPR017853),Glycoside hydrolase, catalytic domain (IPR013781),Glycoside hydrolase family 18, catalytic domain (IPR001223),Chitinase II (IPR011583),chitinase insertion domain (IPR029070)
PEX2_091460	1444	2	43	Glycoside hydrolase, superfamily	Chitin-binding type 1 (IPR001002), Glycoside hydrolase superfamily (IPR017853),Glycoside hydrolase, catalytic domain (IPR013781),Glycoside hydrolase family 18, catalytic domain (IPR001223),Chitinase II (IPR011583),chitinase insertion domain (IPR029070), Ecp2 effector domain (IPR029226)

**Fig 1:**
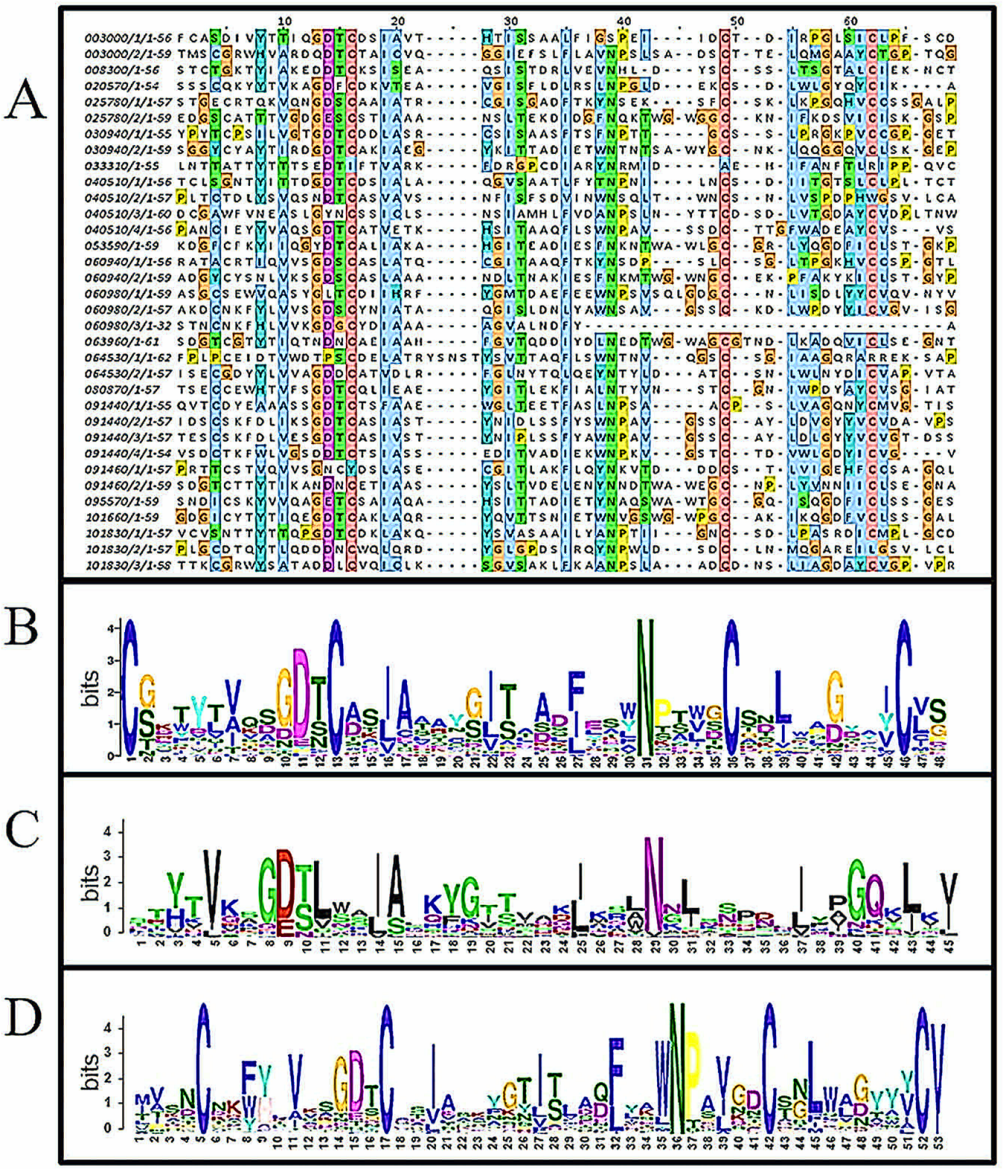
Analysis of LysM domains found in *P*. *expansum* genome. (A) Multiple alignment of all predicted LysM domains found in the genome of *P*.*expansum*. (B) Sequence logo for the multiple alignment. (C) Sequence logo for LysM domain defined by Protein A of *Staphylococcus aureus* (Prosite PS51782). (D) Sequence logo for consensus of fungal-specific LysM motifs [21].

To characterize the LysM consensus pattern typical for *P*. *expansum*, amino acid sequences encoded by the 18 LysM-genes from *P*. *expansum* genome were aligned (Fig 1A). The graphical sequence logo generated using GLAM2 to represent sequence conservation of the *P*. *expansum* nucleotides (Fig 1B) differed from that of the LysM domain profile defined by Protein A of *Staphylococcus aureus* (PROSITE PS51782, Fig 1C). Akcapinar and Kappel (2015) derived a general fungal LysM consensus pattern profile by analyzing more than 900 fungal-specific LysM. The fungal-type LysM consensus pattern containing four conserved cysteine residues is different from the general LysM pattern present in the SMART and PFAM databases. Phylogenetic analysis indicated a rather large evolutionary distance between fungal specific clades containing LysM with multiple cysteines (similar to Tal6) and a group of several clades containing bacterial or bacterial/fungal LysM with one or no cysteine residues, e.g. Ecp6, SLP1, and MgLysM3 [21].

Similar to the consensus pattern constructed by Akcapinar and Kappel (2015) (Fig 1D), the LysM consensus pattern present in the PEX2 genome also contains four conserved cysteine residues and an N in the WNP motif and is likely to belong to a class of fungi-specific LysM. The fungal-specific LysM may be stabilized by two disulfide bridges that can potentially be formed by the four fungal-conserved cysteine residues.

### Four of the *PeLysM* genes are actively transcribed

**Fig 2:**
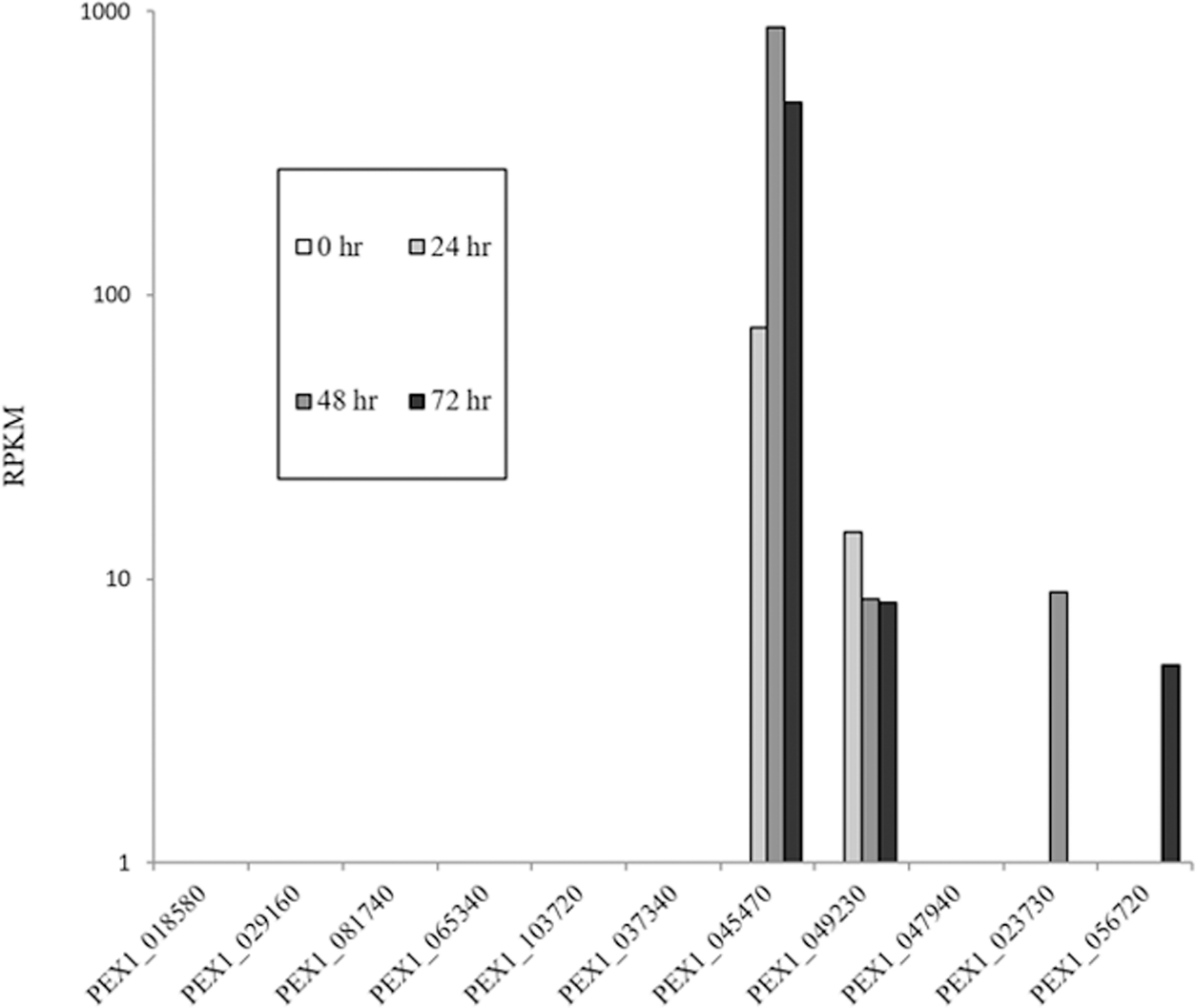
Expression of secreted LysM genes based on the analysis of the RNAseq data of apples infected with PEX1. The RNAseq data was taken from Ballester et al. [41]. RNA-Seq data were generated from four different libraries: a cDNA library synthesized from a mixture of RNAs obtained from *P*. *expansum* PEX1 spores collected after 7 days of growth in PDA and from healthy, non-inoculated fruits, and three libraries from *P*. *expansum*–infected apples at 24, 48, and 72 hpi, respectively. The relative expression of the genes is expressed as RPKM—Reads Per Kilobase of transcript per Million mapped reads.

Our attempt to identify and characterize putative LysM-effectors in *P*.*expansum* focused on eleven LysM proteins with predicted secretory pathway signal peptide Using the RNAseq data generated form the PEX1 isolate during infection of apple [41], we were able to confirm the active transcription of four LysM genes (PEX1_045470, PEX1_049230, PEX1_023730, PEX1_056720) out of eleven LysM with signal peptides during apple infection (Fig 2).

**Fig 3:**
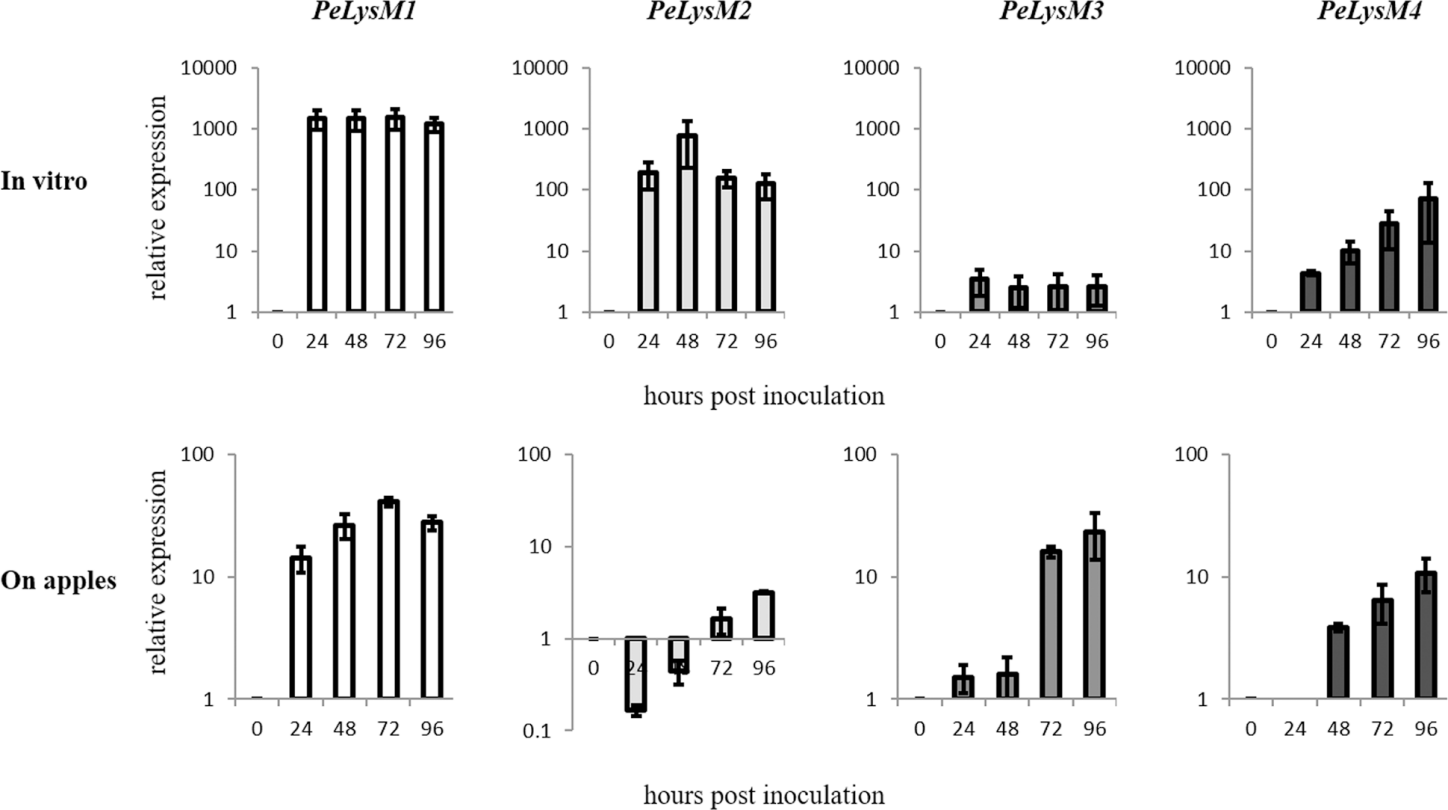
Expression of the *PeLysM1*, *PeLysM2*, *PeLysM3* and *PeLysM4* in PDB culture and during infection and development of *Penicillium expansum* on apple fruit. Relative gene expression was calculated from Cq values using a ΔΔCq method [40]. The results are an average of three independent measurements. Bars indicate standard error.

These genes are identical to PEX2_020570, PEX2_091440, PEX2_053590, and PEX2_064530, respectively, in the PEX2 isolate [41]. The genes were named *PeLysM1*, *PeLysM2*, *PeLysM3* and *PeLysM4* and their expression level in the PEX2 isolate was determined using RT-qPCR. All four genes were actively expressed *in vitro* (PDB culture) and during apple infection (Fig 3). The expression level of each of the four genes increased during the infection of apple, with *PeLysM1* increasing up to 72 hpi and the other three *LysM* genes increasing up to 96 hpi. *Ecp6* from *C*. *fulvum*, as well as *Mg1LysM* and *Mg3LysM* from *M*. *graminicola*, were also reported to be up regulated during the infection of their respective hosts [22, 25]. Interestingly, all of the *LysM* genes, except *PeLysM3*, exhibited a stronger induction when grown *in vitro* (Fig 3). This is in contrast to *Mg1LysM* and *Mg3LysM* that only exhibited pronounced transcriptional activation during wheat leaf infection [25]. The expression of the LysM-containing genes, *tvl1-tvl6*, in the beneficial fungus, *Trichoderma virens*, harvested from mycelia interacting with maize roots challenged with *Cochliobolus heterostrophus* was also shown to be lower than in mycelia grown in media with glucose as carbon source [21].

### Bioinformatics analyses of four putative LysM effectors in *P*. *expansum*

**Fig 4:**
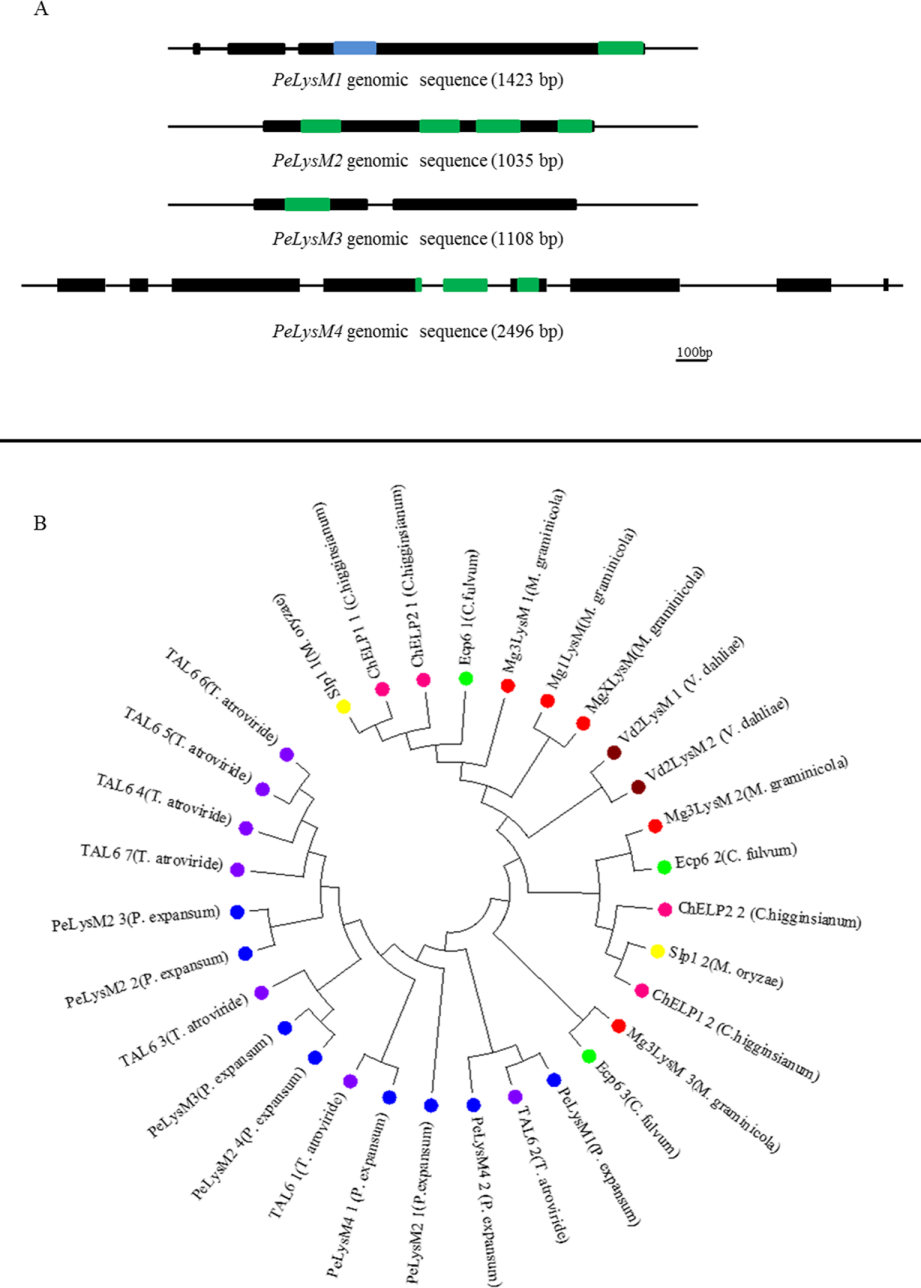
Gene structure and phylogenetic analysis of the four putative PeLysM effectors. (A) Diagrammatic representation of the structures of the four PeLysM genes. Black bars indicate the predicted exons present in the gene model, green rectangles- LysM domains, blue rectangle—lysozyme domain. The genomic sequence length in bp is given for each gene as an indication of scale; (B) Phylogram with distance indicator showing the relatedness of the LysM domains from the four *P*. *expansum* putative effectors to known fungal effector-proteins based on the alignment of individual LysM domains from these proteins. Each color indicates different specie: blue–*P*. *expansum*, purple–*T*. *atroviride*, red–*M*. *graminicola*, green–*C*. *fulvum*, yellow–*M*. *oryzae*, brown–*V*. *dahlia*, pink–*C*. *higginsianum*.

Analysis of the four identified *LysM* genes using PROSITE revealed that PeLysM1 and PeLysM3 have one LysM domain each at the C terminus and N terminus, respectively, while PeLysM4 has two LysM domains, and PeLysM2 has four domains. PeLysM2, PeLysM3, and PeLysM4 do not appear to possess any other conserved domains, however, PeLysM1 also contains a lysozyme-like domain (pfam01464) (Fig 4A). A number of proteins carrying both LysM and lysozyme-like domains have been reported in the literature. Garvey et al. (1986) reported that gene 15 (Gp15) of *Bacillus* phage phi 29 which plays a role in morphogenesis, has a lysozyme-like domain at N-the terminus and two LysM domains in the C-terminus [46]. Two LysM proteins, LSL_0304 and LSL_0805, in *Lactobacillus salivarius* UCC118 were identified as phage-related and both were annotated as a lysozyme [47]. Another LysM protein with a lysozyme domain is the Rpf growth factor of *Mycobacterium luteus*, which was shown to regulate the ability of the bacteria to grow in culture via enzymatic modification of the bacterial cell envelope [48, 49, 50].

In order to identify sequence similarities, the predicted amino acid sequences of each of the LysM domains found in PeLysM1, PeLysM2, PeLysM3, and PeLysM4 were aligned to the LysM domains from known fungal effectors: Ecp6 (*C*. *fulvum*), Mg3LysM, Mg1LysM and MgXLysM (*M*. *graminicola*), Slp1 (*M*. *oryzae*), ChELP1 and ChELP2 (*C*. *higginsianum*), Vd2LysM (*V*. *dahlia*) and LysM domains from TAL6 protein involved in self-regulation of fungal growth (*T*. *atroviride*). A phylogram illustrating the relatedness of the LysM domains was then generated (Fig 4B). In general, LysM domains from *P*. *expansum* were grouped with the LysM domains of the *T*. *atroviride* TAL6 protein, rather than other LysM effector proteins. The putative *P*. *expansum* LysM effectors appear to belong to a clade of fungal-specific proteins containing LysM domains with multiple cysteines (similar to Tal6), rather than to a group of several clades containing either bacterial or mixed bacterial/fungal LysMs, that possess LysM domains with only one or no cysteine, such as Ecp6, Slp1, and MgLysM3 [21].

### Knockouts of *PeLysM* genes

**Fig 5:**
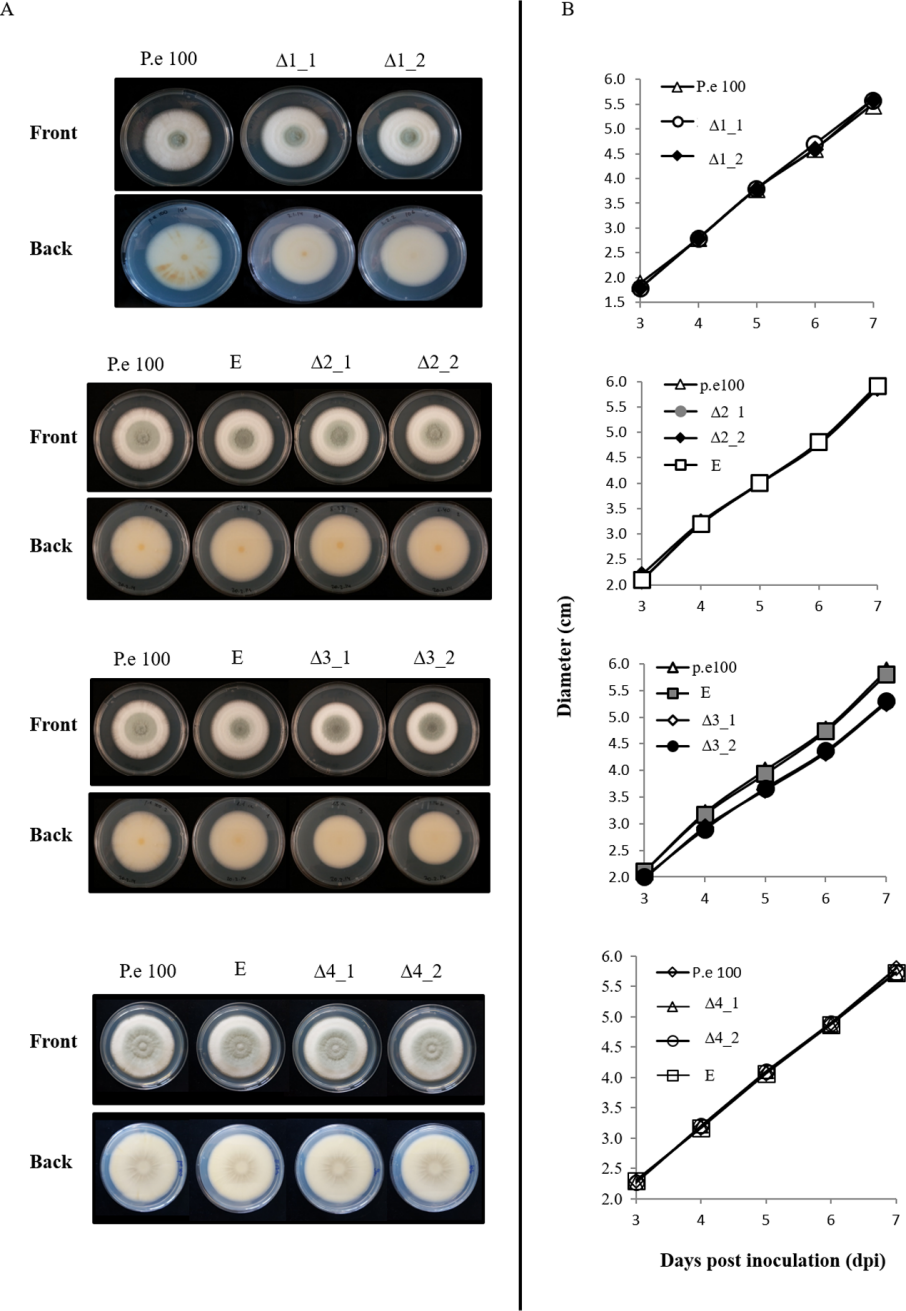
Effect of the targeted deletion on the growth rate and colony morphology. (A) Colony morphology at 7 dpi (B) radial growth on PDA medium of the wild type (P. e 100), ectopic mutants (E), and knockout mutants for *PeLysM1* (Δ1), *PeLysM2* (Δ2), *PeLysM3*(Δ3), and *PeLysM4*(Δ4). Radial growth results are an average of three independent measurements. Bars indicate standard error Statistical analysis present in S2 Table.

**Fig 6:**
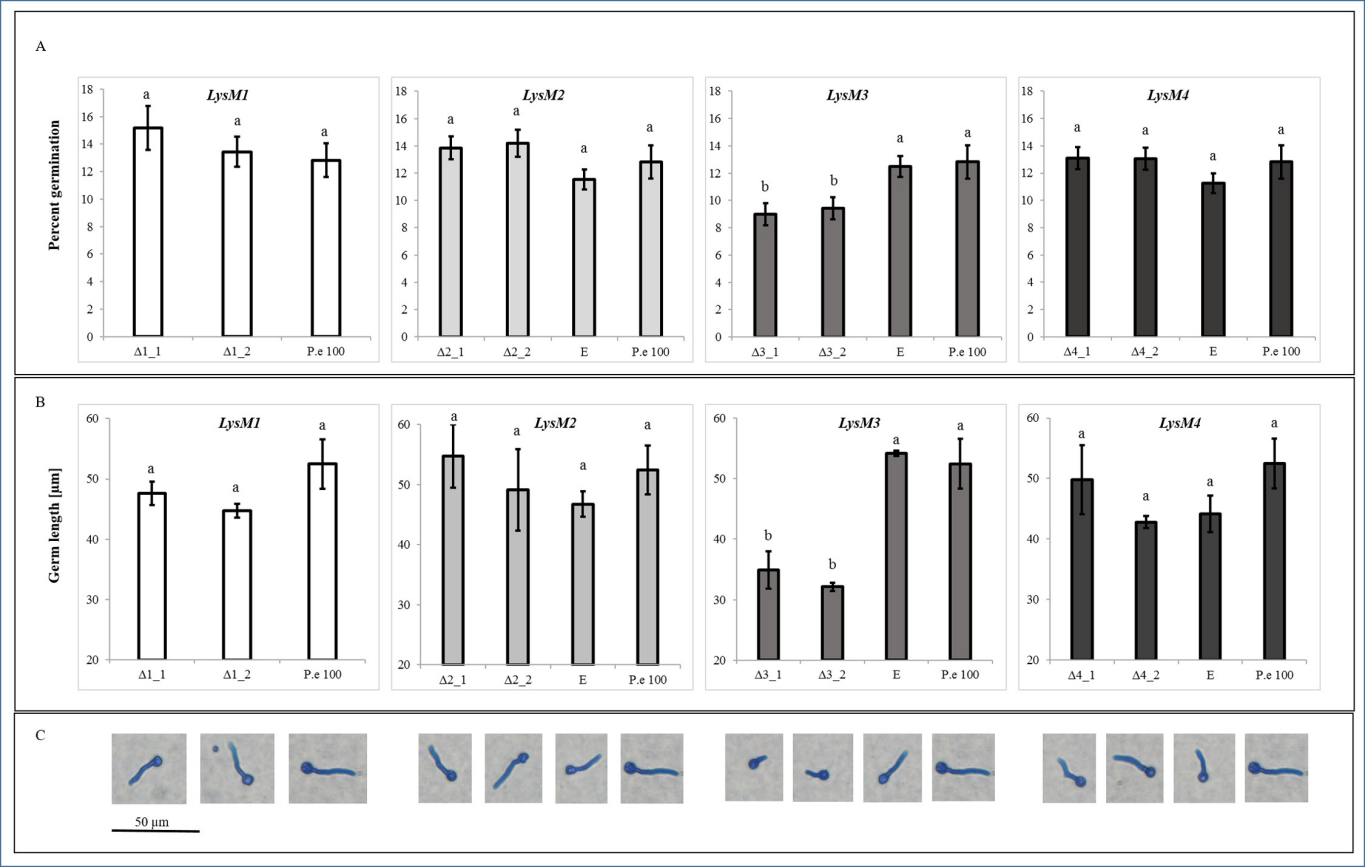
Effect of the target deletion on the germination. (A) Percent of germinating spores, (B) length of the germ tubes and (C) microscope observation of the germination of spore 18 hr post inoculation on 1.5% agar plates. Wild type (P. e 100), ectopic mutants (E), knockout mutants for *PeLysM1* (Δ1), *PeLysM2* (Δ2), *PeLysM3*(Δ3), and *PeLysM4*(Δ4). Percent of germination is an average of three different microscopic fields including 100–120 spores each. Length of the germs is an average of more than 50 measurements. Bars indicate standard error. Different letters indicate significant differences at *P*<0.05 based on nested one-way ANOVA followed by Tukey’s honest significant difference (HSD) test.

The role of PeLysM1, PeLysM2, PeLysM3 and PeLysM4 in pathogenicity of *P*. *expansum* was examined by constructing knockout mutants in PEX2 using *Agrobacterium tumefaciens*–mediated transformation [41]. S1A Fig diagrammatically illustrates the respective wild-type and gene-disrupted loci. Proper T-DNA integration was checked by PCR (S1B Fig) using locus-specific primers for i) the hygromycin resistance cassette which is present in both null mutants and ectopic transformants, but absent in the WT and the target gene, and primers for the target genes, which are absent in the null mutants and present in the WT and ectopic mutants, and ii) correct insertion of the T-DNA between the promoter and the terminator of the target gene. To this end, PCR reaction was done using one primer from the hygromycin resistance cassette and another primer located in the genome approximately 150 bp upstream or downstream of the promoter or terminator respectively. In this case only in null mutants with correct insertion specific PCR product was obtained (S1B Fig). The absence of expression of the deleted genes in the null-mutants during apple infection was demonstrated by RT-PCR for *PeLysM1* and *PeLysM2*, *PeLysM4* and by qRT-PCR for *PeLysM3* (S1C Fig). Two independent knockout mutants, each containing a single T-DNA integration, were selected for further testing, along with an ectopic transformant possessing the wild-type (WT) gene. Deletion of *PeLysM1*, *PeLysM2*, and *PeLysM4* had no effect on radial growth rate and colony morphology of the mutants when they were grown on PDA medium (Fig 5A and 5B). The *PeLysM3* null mutant, however, exhibited slightly lower rate of radial growth compared to the WT strain and the ectopic mutant (S2 Table). Moreover, examination of germination of spores on 1.5% agar plates at 18 hr post inoculation revealed that *PeLysM3* null mutant had significantly lower percent of germinating spores and shorter germ tubes (Fig 6A, 6B and 6C). None of the mutations found to have an effect on spore production (S2 Fig). These findings suggest the possibility that *PeLysM3* has a potential role in growth processes, similar to TAL6 in *T*. *atroviride* which was demonstrated to be involved in self-signaling processes during fungal growth rather than fungal-plant interactions [29].

**Fig 7:**
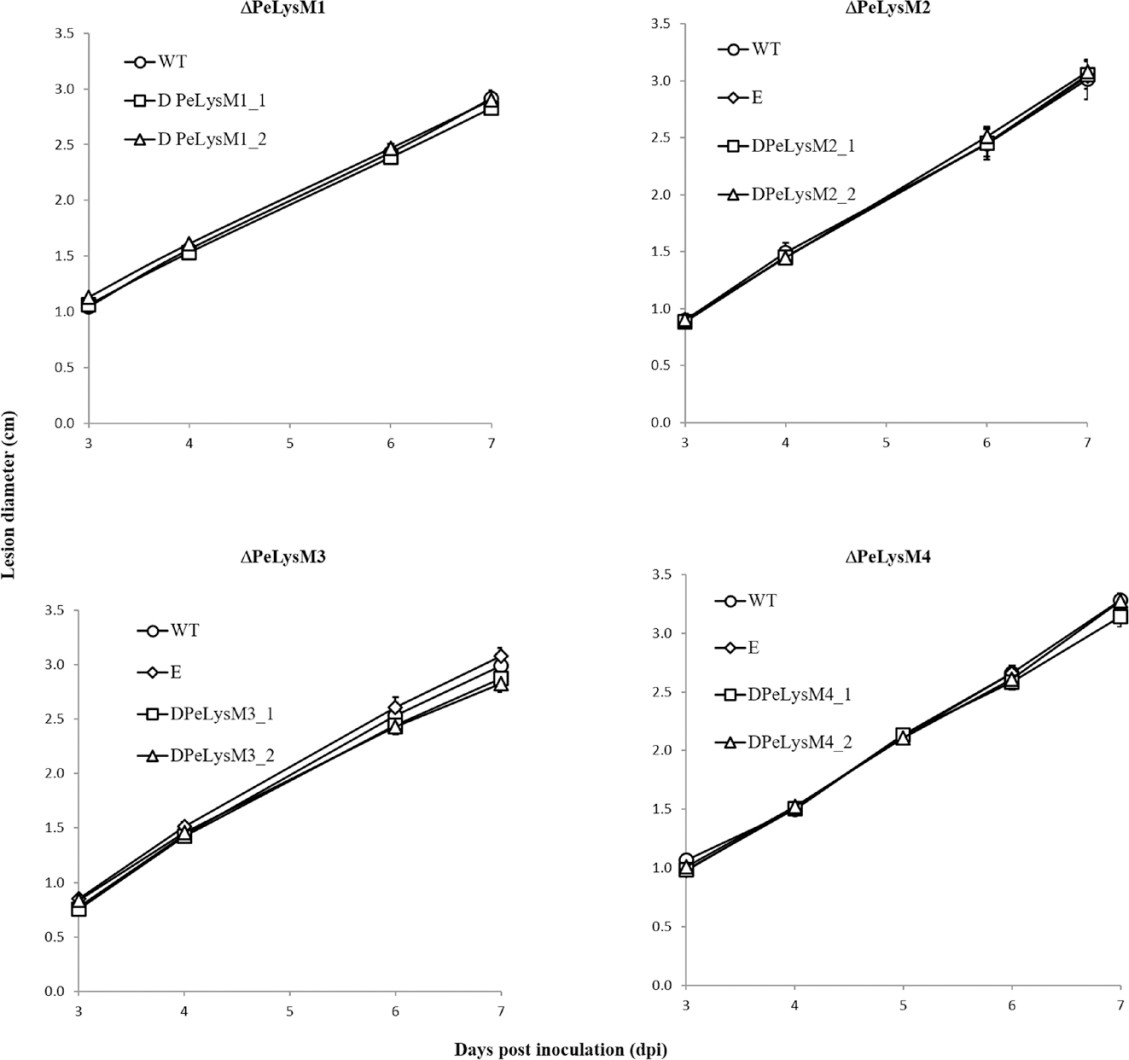
The effect of the deletion on pathogenicity of PEX2 on apples as expressed by rate of decay development on apples and compared to that of the WT and the ectopic mutants. Progression of rot development was expressed as lesion diameter (mm) at different time post inoculation (dpi). Results are an average of three independent measurements. Bars indicate standard error.

To examine the effect of the various deletions on pathogenicity, ‘Golden Delicious’ apples were inoculated with the null mutants. The rate of lesion development and the percent of infected wounds were compared between null mutants, ectopic mutants, and the WT strain. As illustrated in Fig 7, no significant effect of the null mutations on the rate of lesion development was observed relative to the ectopic mutants and WT strain. No significant differences in infection incidence were observed either (not shown). One possible explanation for these results is that other LysM genes present in PEX2 genome could compensate for the separate deletion of any single one of them. In this regard, Mosquera *et al*. (2009) did not see any phenotypic changes in individual knockout mutants of the BIC-associated secreted effectors, BAS1, BAS2, BAS3, and PWL2 from *M*. *oryzae* [51]. In *Verticillium dahlia* strain JR2, targeted deletion of the individual LysM effector genes did not compromise virulence in infections on Arabidopsis, tomato or Nicotiana [28]. As a necrotrophic pathogen with a wide host range, *P*. *expansum* could use an attack strategy that is different from that of *C*. *fulvum*, *M*. *oryzae*, *M*. *graminicola*, *V*. *dahlia and C*. *higginsianum* where LysM effectors were shown to significantly contribute to virulence [15, 25, 26, 27, 28]. Furthermore, the fact that putative LysM effectors are present also in saprophitic fungi indicates that these proteins may be involved in general physiological processes, such as cell wall modification, hyphal growth, branching, morphogenesis, and spore germination [23, 29]. Additionally, LysM proteins were proposed to help fungi to affect bacterial competitors in their niches, protect fungal hyphae from chitinases secreted by competitor microbes or decrease the attraction of mycoparasites. by the sequestration of chitin oligosaccharides [20, 23].

### Identification of apple proteins interacting with *P*. *expansum* LysM effectors

Four LysM-coding genes were identified in the *P*. *expansum* genome that are actively transcribed during infection and decay development on apple and predicted to be secreted. The deletion of three out of four LysM coding genes had no effect on pathogenicity and virulence or on growth. Therefore, a yeast two-hybrid (Y2H) system was used to gain insight into the potential interaction of the secreted LysM proteins with apple proteins. The LysM domain (240 nt) from *PeLysM1* was amplified by PCR, fused with the GAL4 DNA binding domain in the pGBKT7 vector and used as a bait. The prey library was constructed from total RNA isolated from ‘Royal Gala’ apple fruit collected at three different stages of development (early, mid-season, and mature). Tissue was collected from fruit that were either left unwounded, wounded, or wounded-inoculated with *P*. *expansum*. RNA was extracted from all three types of fruit tissues and three conditions and pooled prior to generating cDNA. The library was packaged into the prey vector fused to the GAL4 activation domain. Several (3–4) rounds of screening were conducted in order to reduce the potential of false positives. Positive clones were verified by inserting the individual positive clones into the Y2H system to serve as prey, conducting the mating again, followed by subsequent growth on highly selective medium (S3 Fig). In some cases, several of the identified clones represented different portions of the same gene. BLAST results where the sequenced clones were used as queries against the *M*. *× domestica* genome resulted in the identification of 37 genes (Table 3). None of the identified genes are present in clusters and are instead distributed throughout the apple genome on several chromosomes. Analysis of the identified genes with SignalP indicated that three of the genes (MDP0000157048, MDP0000218404, MDP0000576346) possess a predicted signal peptide. Analysis by TargetP indicated that MDP0000157048 is targeted to the chloroplast, while the other two are predicted to be secreted. One of the secreted proteins (MDP0000218404) is a cysteine-peptidase (C1A). Several studies have been reported on the role of cycteine-peptidase in plant defense against insects and fungi [52, 53]. One of the most relevant examples is RCR3, a papain-like protease (C1) from tomato. RCR3 is required for the function of *Cf-2*, a resistance gene that mediates recognition of *Avr2*. RCR3 contains a granulin domain, which in plants is often located in the C terminus of cysteine proteases [54]. A second putative secreted apple protein (MDP0000576346) is similar to an avirulence gene for the fungal pathogen, *Cladosporium fulvum* [55]. Another identified protein, MDP0000586674, that belongs to the peptidase family C1 (subfamily C1A –papain), is cytochrome P450, which in plants has been shown to play a significant role in the biosynthesis of several plant metabolites, including hormones, defensive compounds, and fatty acids [56, 57]. Both of the secreted proteins could potentially play an important role in the defense of fruit against *P*. *expansum* and are excellent candidates for further future study.

**Table 3:** Apple gene- products that interacted with LysM.

#		Gene ID	# hits	Exp	Align Length	Chrom.	Function	S*	T**
1		MDP0000121897	1	1e-104	193	8	Phosphoribosyltransferase		
2		MDP0000129126	1	1e-134	246	1	BAG domain, Ubiquitin supergroup		
3		MDP0000137468	1	1e-154	283	1	Late embryogenesis abundant protein, LEA-14		
4		MDP0000157048	2	4e-87; 0.0	174; 547	15	Polyketide synthase, Thiolase-like, 3-oxoacyl-[acyl-carrier-protein] synthase 2	+	C
5		MDP0000177180	1	8e-21	51	4	Peptidyl-prolyl cis-trans isomerase, PpiC-type		
6		MDP0000201315	1	1e-178	316	4	Peptidyl-prolyl cis-trans isomerase, FKBP-type		
7		MDP0000208334	1	7e-17	61	10	bZIP transcription factor		C
8		MDP0000218404	1	0.0	353	2	Peptidase C1A, papain	+	S
9		MDP0000219010	1	0.0	393	9	Protein synthesis factor, GTP-binding, Translation elongation/initiation factor/Ribosomal		
10		MDP0000232800	1	0.0	334	15	SWIB/MDM2 domain, GYF, Plus-3		C
11		MDP0000253306	1	1e-115	286	6	Clathrin adaptor, mu subunit, Longin-like, AP complex		
12		MDP0000265644	1	0.0	481	10	Oxoglutarate/iron-dependent oxygenase		
13		MDP0000271244	1	4e-19	52	13	Ribose 5-phosphate isomerase, type A		
14		MDP0000277802	1	1e-134	241	unanchored	Bet v I domain, START-like domain, Major latex protein domain		
15		MDP0000286750	1	0.0	475	12	Ferritin		
16		MDP0000290323	1	2e-25	63	10	CDC45 family, Pathogenic type III effector avirulence factor Avr cleavage site		
17		MDP0000295540	3	0.0; 2e-13; 0.0	480; 57; 480	13	START-like domain, Bet v I type allergen		
18		MDP0000299239	1	3e-55	109	13	Ribosomal protein S21e, Threonyl/alanyl tRNA synthetase, SAD		
19		MDP0000303994	1	2e-49	99	3	Snf7		
20		MDP0000329229	1	1e-118	217	14	Fructose-bisphosphate aldolase, class-I		
21		MDP0000335264	1	1e-174	323	9	Orotidine 5'-phosphate decarboxylase domain, Ribulose-phosphate binding barrel, Aldolase-type TIM barrel		
22		MDP0000341606	1	1e-174	317	4	Protein-tyrosine/Dual-specificity phosphatase		
23		MDP0000360447	1	0.006	21	6	Oxoglutarate/iron-dependent oxygenase		
24		MDP0000361244	1	1e-103	192	14	Ribosomal protein S3, bacterial		
25		MDP0000415257	1	0.0	495	16	Universal stress protein A, Rossmann-like alpha/beta/alpha sandwich fold		
26		MDP0000478750	1	1e-118	221	5	Tyrosinase, Twin-arginine translocation pathway, signal sequence, Polyphenol oxidase.		C
27		MDP0000576346	1	0.0	421	3	Cytochrome P450	+	S
28		MDP0000586674	1	1e-126	249	8	Granulin, Peptidase C1A, papain		
29		MDP0000607509	1	1e-117	221	5	Polyphenol oxidase		
30		MDP0000645828	1	0.0	417	16	Glyceraldehyde-3-phosphate dehydrogenase, type I		
31		MDP0000659790	1	0.0	404	3	Domain of unknown function DUF303, acetylesterase putative, Esterase, SGNH hydrolase-type		
32		MDP0000699845	2	1e-144; 1e-162	295; 305	10	Tyrosinase, Polyphenol oxidase		C
33		MDP0000704686	1	2e-09	32	15	Short-chain dehydrogenase/reductase SDR, Glucose/ribitol dehydrogenase		
34		MDP0000708928	1	1e-129	313	9	Chlorophyll A-B binding protein		
35		MDP0000776395	1	0.0	350	14	C2 membrane targeting protein		
36		MDP0000827820	2	1e-123; 1e-125	276; 246	13	Bet v I type allergen, START-like domain		
37		MDP0000872167	2	1e-153; 0.0	292; 423	12	Late embryogenesis abundant protein, LEA-14		

*SignalP

**TargetP

TargetP analysis identified another four proteins (MDP0000208334, MDP0000699845, MDP0000232800, MDP0000478750) that are targeted to the chloroplast. (Two (MDP0000699845, MDP0000478750) are tyrosinases, which catalyze the hydroxylation of monophenols and the oxidation of o-diphenols to o-quinols. These enzymes are involved in the formation of pigments, such as melanins and other polyphenolic compounds. Other proteins that belong to this family are plant polyphenol oxidases (PPO) (EC:1.10.3.1), which catalyze the oxidation of mono- and o-diphenols to o-diquinones. Plant PPO genes are nuclear encoded and are generally targeted to plastids. PPOs appear play a role in biotic and abiotic stress responses [58]. These enzymes are generally considered to function in damaged tissues, where mechanisms including quinone toxicity, a reduction in the nutritive value of proteins, and the formation of structural barriers can offer protection against pathogens [59]. Another PPO gene, MDP0000607509 that is not targeted to chloroplast was also identified. It is a plant type PPO with a twin-arginine translocation (Tat) pathway signal sequence.

Another three chloroplast-predicted proteins that were identified are: MDP0000208334, which contains a basic-leucine zipper domain that could be involved in transcription regulation; and MDP0000232800 which contains a SWIB/MDM2 domain, that may be involved in chromatin-remodeling that facilitates transcription activation [60]. MDP0000157048 is a beta-ketoacyl synthase. The main function of this protein is in the synthesis of fatty acids. Beta-ketoacyl-ACP synthase is found as a component of a number of enzymatic systems, including the multi-functional 6-methysalicylic acid synthase (MSAS) in *Penicillium* *patulum*, which is involved in the biosynthesis of a polyketide antibiotic [61], and in *Rhizobium* nodulation protein nodE, which probably acts as a beta-ketoacyl synthase in the synthesis of the nodulation Nod factor fatty acyl chain [62]. Interestingly, the Nod factor contains an acylated chitin oligomeric backbone that is recognized by plant LysM receptor kinases [63, 64].

In addition to cytochrome P450 and the PPO genes that were already mentioned, four additional genes were identified in the Y2H screen, which encode proteins involved in oxidation-reduction processes. These included two oxoglutarate/iron-dependent oxygenases (MDP0000265644, MDP0000360447), known to catalyze the formation of plant hormones, such as ethylene, gibberellins, anthocyanidins, and pigments such as flavones [65]. MDP0000645828 is a glyceraldehyde-3-phosphate dehydrogenase (GAPDH), type I. In addition to its role in glycolysis, GAPDH has been reported to have diverse, non-glycolytic functions depending upon its subcellular location. For instance, the translocation of GAPDH to the nucleus acts as a signaling mechanism for programmed cell death [66]. Another gene coding for redox protein that was identified is MDP0000704686, a short-chain dehydrogenase/reductase SDR, that is a member of a large family of enzymes, most of which are known to be NAD- or NADP-dependent oxidoreductases.

Additionally, three proteins (MDP0000232800, MDP0000295540, and MDP0000827820) with Bet v I and START-like domains were identified. Bet v 1 belongs to family 10 of plant pathogenesis-related proteins (PR-10). These proteins were identified as both plant allergens and as pathogenesis-related proteins whose expression is induced by pathogen infection, wounding, or abiotic stress. They appear to be involved in pathogen defense response [67, 68].

Several additional proteins that could potentially play a significant role in the fruit-pathogen interaction were also identified: MDP0000415257, universal stress protein A; and MDP0000129126 that possesses both a BAG domain and an ubiquitin domain. BAG-1 has been suggested to function as a molecular switch that encourages cells to proliferate in normal conditions but become quiescent when exposed to a stressful environment [69]. MDP0000341606 is a protein-tyrosine/dual-specificity phosphatase that can function as one of the key regulatory components in signal transduction pathways (such as the MAP kinase pathway). MDP0000290323, is a member of the cell division control protein 45 (CDC45) family, that are essential genes required for the initiation of DNA replication in *Saccharomyces cerevisiae* [70]. MDP0000286750 contains a ferritin domain that is present in proteins reported to accumulate in transgenic plants whose leaves exhibit tolerance to necrotic damage caused by the fungi *Alternaria alternata* and *Botrytis cinerea* [71]. Interestingly, Potrykus *et al*. (2013) who demonstrated the first case of systemic nutritional immunity against a fungal infection, found that when *C*. *albicans* formed lesions in the renal cortex (a pathology typical for this form of candidiasis), iron was mobilized away from the fungal lesions to the renal medulla [72]. This event was associated with increased levels of the host iron-binding proteins, ferritin and haemoglobin alpha in the medulla of infected kidneys, indicating that the sequestration of iron away from the sites of infection is indeed a coordinated host-driven process.

Two of the identified proteins appear to be involved in protein folding. Peptidyl-prolyl cis-trans isomerases MDP0000201315 is a FKBP-type, and MDP0000177180 is a PpiC-type peptidyl-prolyl cis-trans isomerase. Another two proteins (MDP0000121897 and MDP0000335264) are involved in nucleotide metabolic pathways and an additional two (MDP0000303994, MDP0000253306) may play a role in intracellular trafficking. Lastly, three ribosomal proteins wereidentified: MDP0000299239 a ribosomal protein S21e, MDP0000361244 a ribosomal protein S3, and MDP0000219010 a translation elongation factor EF1A/initiation factor IF2gamma. Though the role of these proteins in host-pathogen interactions is not readily apparent, it is important to note that many ribosomal proteins have a function 'outside' of the ribosome.

Lys-M effectors are generally known to bind carbohydrate residues, such as chitin moieties, presumably so that they will not be recognized by host tissues and induce a resistance response [23]. To the best of our knowledge, the interaction of LysM with host proteins has not been previously reported. Understanding the basis and role of these potential protein-protein interactions, however, is challenging. In this regard, motif analysis of LysM proteins from *P*. *expansum* as well as some other known fungal LysM effectors in SMART revealed several regions of low structural complexity. The flexible nature of these regions are believed to be responsible for their versatile binding capabilities; this flexibility could allow these regions to bind several different targets [73, 74]. The portion of the *P*. *expansum* domain that was used in the Y2H experiment also contained such region. Interestingly, many of the proteins that were identified in the Y2H study are connected to host defense mechanisms, either as PR proteins or defense related molecules, such as polyphenol oxidases. One can speculate that LysM proteins are secreted by *P*. *expansum* to interfere with PTI by binding to chitin oligomers, but then recognized by the host proteins and elicit ETI. In this regard, Kombrink et al. (2013) have noted that while the function of several LysM fungal proteins has been identified, the role of many remains obscure. Therefore, while nothing definitive can be said about the function of the LysM domain from *P*. *exapansum*, or the basis of the interaction with the identified apple proteins, the stringency and re-mating confirmation conditions used in the Y2H study, suggest that the interactions were real and should be explored in more detail in future studies.

## Conclusions

The results presented in this study provide the first information on the existence of 18 putative genes in *P*. *expansum* genome encoding secreted and non-secreted LysM proteins. Among the identified LysM genes, 4 were found to be highly expressed during the infection and development of decay of apple fruit. Amino acid sequence similarities were found between these proteins and the closely-related *T*. *atroviride* LysM proteins, indicating that *P*. *expansum* LysM proteins belong to a clade of fungal-specific proteins containing multiple LysM domains. Functional analysis of the *LysM* genes revealed no significant effect on the pathogenicity and virulence of *P*. *expansum* on apples when each of the four genes encoding LysM effectors proteins were knocked out separately. These findings suggest that if pathogenicity and virulence of *P*. *expansum* on apple is associated with the LysM effector proteins, they act in concert with each other during infection and decay development and the loss of one of them can be compensated by the remaining three. Multiple knockouts will be required to test this premise. A slight reduction in spore germination and growth rate, however, was found when *PeLysM3* was deleted. This result, along with the similarity of *P*. *expansum* LysM to TAL6 of *T*. *atroviride*, suggests that some of LysM proteins could play a role in fungal growth and development. Alternatively, a possible role of *P*. *expansum* LysM effector proteins in binding chitin oligomers and or protection of fungal hyphae from competing microorganisms should be considered and warrants future study.

To obtain further preliminary insights on possible potential role of *P*. *expansum* putative LysM effector proteins in the interaction with the host tissue during infection, Y2H studies utilizing the LysM domain as a bait, identified a wide range of interacting apple proteins with diverse functions, including plant defense processes and iron sequestration, further highlighting the possibility that *P*. *expansum* LysM effector proteins may play potential role modulating cellular processes in host tissue in the early stages of infection and subsequent development of decay. These findings may serve as the basis of future studies to understand the role of *P*. *expansum* LysM effector proteins in modulation of host defenses during early stages of the infection and decay development process.

## Supporting information

S1 Table Primers used in the study. (DOCX)

S2 Table Statistic analysis of radial growth of *ΔPeLysM3* null mutants (*Δ*3) compared to WT (P. e 100) and ectopic mutant (E). (DOCX)

S3 Table Statistic analysis of *ΔPeLysM1* pathogenicity test. (DOCX)

S4 Table Statistic analysis of *ΔPeLysM2* pathogenicity test. (DOCX)

S5 Table Statistic analysis of *ΔPeLysM3* pathogenicity test. (DOCX)

S6 Table Statistic analysis of *ΔPeLysM4* pathogenicity test. (DOCX)

S1 Fig Deletion of PeLysM1, PeLysM2, PeLysM3 and PeLysM4 in *Penicillium expansum* PE100. (A) Diagram of the wild type and deleted PeLysM1, PeLysM2, PeLysM3 and PeLysM4 loci. The hygromycin selectable marker present in the T-DNA of plasmids pRFHU2-PeLysM1, pRFHU2-PeLysM2, pRFHU2-PeLysM3 and pRFHU2-PeLysM4 replaces the corresponding *PeLysM* genes by homologous recombination, to generate the *ΔPeLysM1*, *ΔPeLysM2*, *ΔPeLysM3* and *ΔPeLysM4* null mutants, respectively; (B) PCR amplification of the wild type, *ΔPeLysM1*, *ΔPeLysM2*, *ΔPeLysM3* and *ΔPeLysM4* null mutants and their respective ectopic mutants with diagnostic primers; (C) RT-PCR for *ΔPeLysM1*, *ΔPeLysM2*, *ΔPeLysM4* or qRT-PCR for *ΔPeLysM3* to compare the expression of LysM genes in wild type (P.e100), null (Δ) and ectopic (E) mutants in apples. 37S ribosomal protein s24 was used as a control. RGE–relative gene rxpression was calculated from Cq values using a ΔΔCq method [38]. Arrows in figures indicate the primers used. Arrows connected with line shows expected PCR product. Original gel images are available in S1 Appendix.
(DOCX)

S2 Fig Effect of targeted deletion of *PeLysM1*, *PeLysM2*, *PeLysM3* and *PeLysM4* on spore production. Average number of spore produced after 11 days of growth on potato dextrose agar medium based on three independent experiments. Bars indicate standard error. Letters indicate significant differences at *P*<0.05 based on nested one-way ANOVA followed by Tukey’s honest significant difference (HSD) test.
(DOCX)

S3 Fig Partial results of the yeast-two hybrid analysis of the interactions between LysM from *P*. *expansum* and apple proteins. Five out 37 of positive clones are presented. The LysM domain (240 nt) from *PeLysM1* was amplified by PCR, fused with the GAL4 DNA binding domain in the pGBKT7 vector and used as a bait. Empty vector was used as negative control. The prey library was constructed from total RNA isolated from ‘Royal Gala’ apple fruit collected at three different stages of development (early, mid-season, and mature). The library was packaged into the prey vector fused to the GAL4 activation domain. The candidate yeast cells were re-streaked several (3–4) times on SD/-Leu-Trp (DDO)/X selective medium. Each time a single blue colony, indicating a positive interaction (blue), was picked for re-streaking. All positive interactions (clones) were confirmed by patching on high stringency screening medium (SD/-Ade-His-Leu-Trp (QDO)/X/A). In order to further confirm that the interactions were genuine, prey plasmids from *E*. *coli* were transformed into the Y2H yeast strain containing the bait plasmid and grown on selective medium QDO/X/A.
(DOCX)

S1 Appendix Original gel images. (PPTX)
