# Supplementary material for: Identification and characterization of LysM effectors in Penicillium expansum
Source: PLoS One. 2017 Oct 30;12(10):e0186023. doi: 10.1371/journal.pone.0186023 (PMC5662087; doi:10.1371/journal.pone.0186023)
Supplement: S1 Table — (DOCX) [file pone.0186023.s001.docx]

**S1 Table: Primers used in the study.**

| **number** |  | | **Name** | **Sequence** |
| --- | --- | --- | --- | --- |
|  | | **Knockout mutant analysis** | | |
| 1 |  | | LysM1_center_F | GCAATAAGGACGGTAGCAAA |
| 2 |  | | LysM1_center_R | AGAGGTGGTCTCAGTGGATGT |
| 3 |  | | HMBF1 | CTGTCGAGAAGTTTCTGATCG |
| 4 |  | | HMBR1 | CTGATAGAGTTGGTCAAGACC |
| 5 |  | | LysM1_checkF | CCGCTGAGCCTTCGATCCTTATT |
| 6 |  | | HPH1F | ACGAGGTCGCCAACATCTTCTTCT |
| 7 |  | | HPHPRO4 | GCACCAAGCAGCAGATGATA |
| 8 |  | | LysM1_checkR | TGGAAGAAGCACAGACAGCA |
| 9 |  | | LysM2_center_F | CGCAGACATAGTAGCCCA |
| 10 |  | | LysM2_center_R | CTCTTCATCTTCCACCACC |
| 11 |  | | LysM2_check_F | AGGAAGGAAAAAAATGAGCG |
| 12 |  | | LysM2_checck_R | CGCAGACATAGTAGCCCA |
| 13 |  | | LysM3_center_F | ACGGCTATACCAGGGTGAC |
| 14 |  | | LysM3_center_R | GTTGGGGTTGGAGGTAGG |
| 15 |  | | LysM3_check_F | CGTTACAAGGAGCACTGGTCATCG |
| 16 |  | | LysM3_check_R | CTCCGTCTTTGTCAGAGCGT |
| 17 |  | | LysM4_center_F | TGACGAACTTGCTACGAGAT |
| 18 |  | | LysM4_center_R | AGACTGTCTGAGCGGTAACA |
| 19 |  | | LysM4_check_F | GCGTATTCGTTGCATCATTGAAAGAC |
| 20 |  | | LysM4_check_R | AGAGCAATTTGTCCGCATG |
|  | | **Determination of T-DNA copy number** | | |
| 21 |  | | PeTub_1F | AGCGGTGACAAGTACGTTCC |
| 22 |  | | PeTub_1R | ACCCTTAGCCCAGTTGTTAC |
| 23 |  | | LysM1_RT_F | CTTCACAATGACATCGTGTTCG |
| 24 |  | | LysM1_RT_R | GTAATGACCACGTCCAACCCT |
| 25 |  | | LysM2_RT_F | TCTGTAGCTGAGGAGTGTGGAGA |
| 26 |  | | LysM2_RT_R | CGAAGGGTGGGTTTGATAGTG |
| 27 |  | | LysM3_RT_F | GCTTGTTGTCGGACTCCATCT |
| 28 |  | | LysM3_RT_R | TGGTTGTAGGCTGGCAGTTCT |
| 29 |  | | LysM4_RT_F | CTCCTTGGGACCCTTTCACA |
| 30 |  | | LysM4_RT_R | GCCCTTGGGATACGATGCT |
|  | | **Knock out mutant construction** | | |
| 31 |  | | LysM1_Prom_F | GGTCTTAAUGGACGGAGTAGTCATTGG |
| 32 |  | | LysM1_Prom_R | GGCATTAAUTGAGTTGATGATGGCGTT |
| 33 |  | | LysM1_Ter_F | GGACTTAAUGTTCTCTGGCAATTCTCGT |
| 34 |  | | LysM1_Ter_R | GGGTTTAAUTGTAATCTCGCTGTCCGT |
| 35 |  | | LysM2_Prom_F | GGTCTTAAUATGGGCTAGTTGCATCGTA |
| 36 |  | | LysM2_Prom_R | GGCATTAAUGCTTCCCGTCTGTCTTGT |
| 37 |  | | LysM2_Ter_F | GGACTTAAUTCGTCTGGTTGTTCGTTT |
| 38 |  | | LysM2_Ter_R | GGGTTTAAUCTTCTTCGCAACTCTTCATT |
| 39 |  | | LysM3_Prom_F | GGTCTTAAUGGGATGGGAGGACGAAATGG |
| 40 |  | | LysM3_Prom_R | GGCATTAAUGGCTGAAACAAAGATTCGGGG |
| 41 |  | | LysM3_Ter_F | GGACTTAAUCCAGCCCCCTCAATCCAATG |
| 42 |  | | LysM3_Ter_R | GGGTTTAAUTGACGTTGAAACAGCAAAGCA |
| 43 |  | | LysM4_Prom_F | GGTCTTAAUGAATGAGAAGGCAAAATTGATATG |
| 44 |  | | LysM4_Prom_R | GGCATTAAUGACCTGCGTAATCTCTGATGC |
| 45 |  | | LysM4_Ter_F | GGACTTAAUGACTGATCTGAGCATGAGCA |
| 46 |  | | LysM4_Ter_R | GGGTTTAAUGCTACAGGAGAAGTTGGGAA |
| 47 |  | | RF-1 | AAATTTTGTGCTCACCGCCTGGAC |
| 48 |  | | RF-6 | ACGCCAGGGTTTTCCCAGTC |
| 49 |  | | RF-2 | TCTCCTTGCATGCACCATTCCTTG |
| 50 |  | | RF-5 | GTTTGCAGGGCCATAGAC |
|  | | **Gene expression analysis** | | |
| 51 |  | | LysM1_F | GCAAGA AAGTAAGGGCTGCGT |
| 52 |  | | LysM1_R | CCATCGTGGCTTTGCATG |
| 53 |  | | LysM2_F | ACGGTCGCTGATATCGAGAAG |
| 54 |  | | LysM2_R | CACACAGATATAATCGCCGAGC |
| 55 |  | | LysM3_F | GAC CGATGATGGCTTCACGT |
| 56 |  | | LysM3_R | GGCAGAGCA ATACCCATTCTTG |
| 57 |  | | 28S_F | GGAACGGGACGTCATAGAGG |
| 58 |  | | 28S_R | AGAGCTGCATTCCCAAACAAC |
| 59 |  | | 37S_F | GCTCTGGTCTACGACTCCTC |
| 60 |  | | 37S_R | GGAAGCCTTCTCGATCTTGC |
| 61 |  | | His3_F2 | TCTCCGCTTCCAGTCCTCTG |
| 62 |  | | His3_R2 | TTGGTGTCCTCGAAGAGAGAGAC |
|  | | **Yeast two hybrid** | | |
| 63 |  | | LysM1complete_F | ATGTTCTTTCCTTCATTGATCC |
| 64 |  | | LysM1complete_R | TTAAGCCTTGATACAGTACTGAT |
| 65 |  | | LYS domain F | CATGGAGGCCGAATTCTCTGCGCCAGCTGCAACAACTGCA |
| 66 |  | | LYS domain R | GCAGGTCGACGGATCCTTAAGCCTTGATACAGTACTGATA |
