# Supplementary material for: Identification and characterization of LysM effectors in Penicillium expansum
Source: PLoS One. 2017 Oct 30;12(10):e0186023. doi: 10.1371/journal.pone.0186023 (PMC5662087; doi:10.1371/journal.pone.0186023)
Supplement: S2 Table — (DOCX) [file pone.0186023.s002.docx]

**S2 Table: Statistic analysis of radial growth of *∆PeLysM3* null mutants (*∆*3) compared to WT (P. e 100) and ectopic mutant (E).**

| **dpi** | **strain** | **HSD significant letter** |
| --- | --- | --- |
| 3 | P.e100 | I |
|  | Ectopic | I |
|  | *∆PeLysM3_1* | I |
|  | *∆PeLysM*3_2 | I |
| 4 | P.e100 | G |
|  | Ectopic | G |
|  | *∆PeLysM3_1* | H |
|  | *∆PeLysM*3_2 | H |
| 5 | P.e100 | E |
|  | Ectopic | E |
|  | *∆PeLysM3_1* | F |
|  | *∆PeLysM*3_2 | F |
| 6 | P.e100 | C |
|  | Ectopic | C |
|  | *∆PeLysM3_1* | D |
|  | *∆PeLysM*3_2 | D |
| 7 | P.e100 | A |
|  | Ectopic | A |
|  | *∆PeLysM3_1* | B |
|  | *∆PeLysM*3_2 | B |

Letters are indicating significant differences at *P*<0.05 based on nested one-way ANOVA followed by Tukey’s honest significant difference (HSD) test.
