# Supplementary material for: Identification and characterization of LysM effectors in Penicillium expansum
Source: PLoS One. 2017 Oct 30;12(10):e0186023. doi: 10.1371/journal.pone.0186023 (PMC5662087; doi:10.1371/journal.pone.0186023)
Supplement: S5 Table — (DOCX) [file pone.0186023.s005.docx]

**S5 Table: Statistic analysis of *∆PeLysM3* pathogenicity test.**

| **dpi** | **strain** | **HSD significant letter** |
| --- | --- | --- |
| 3 | WT | F |
|  | Ectopic | F |
|  | *∆PeLysM3_1* | F |
|  | *∆PeLysM3_2* | F |
| 4 | WT | E |
|  | Ectopic | E |
|  | *∆PeLysM3_1* | E |
|  | *∆PeLysM3_2* | E |
| 6 | WT | D |
|  | Ectopic | CD |
|  | *∆PeLysM3_1* | D |
|  | *∆PeLysM3_2* | D |
| 7 | WT | AB |
|  | Ectopic | A |
|  | *∆PeLysM3_1* | AB |
|  | *∆PeLysM3_2* | BC |

Letters are indicating significant differences at *P*<0.05 based on nested one-way ANOVA followed by Tukey’s honest significant difference (HSD) test.
