# Supplementary material for: Identification and characterization of LysM effectors in Penicillium expansum
Source: PLoS One. 2017 Oct 30;12(10):e0186023. doi: 10.1371/journal.pone.0186023 (PMC5662087; doi:10.1371/journal.pone.0186023)
Supplement: S6 Table — (DOCX) [file pone.0186023.s006.docx]

**S6 Table: Statistic analysis of *∆PeLysM4* pathogenicity test.**

| **dpi** | **strain** | **HSD significant letter** |
| --- | --- | --- |
| 3 | WT | D |
|  | Ectopic | D |
|  | *∆PeLysM4_1* | D |
|  | *∆PeLysM4_2* | D |
| 4 | WT | C |
|  | Ectopic | C |
|  | *∆PeLysM4_1* | C |
|  | *∆PeLysM4_2* | C |
| 6 | WT | B |
|  | Ectopic | B |
|  | *∆PeLysM4_1* | B |
|  | *∆PeLysM4_2* | B |
| 7 | WT | A |
|  | Ectopic | A |
|  | *∆PeLysM4_1* | A |
|  | *∆PeLysM4_2* | A |

Letters are indicating significant differences at *P*<0.05 based on nested one-way ANOVA followed by Tukey’s honest significant difference (HSD) test.
