# Supplementary material for: Identification and characterization of LysM effectors in Penicillium expansum
Source: PLoS One. 2017 Oct 30;12(10):e0186023. doi: 10.1371/journal.pone.0186023 (PMC5662087; doi:10.1371/journal.pone.0186023)
Supplement: S1 Fig — (A) Diagram of the wild type and deleted PeLysM1, PeLysM2, PeLysM3 and PeLysM4 loci. The hygromycin selectable marker present in the T-DNA of plasmids pRFHU2-PeLysM1, pRFHU2-PeLysM2, pRFHU2-PeLysM3 and pRFHU2-PeLysM4 replaces the corresponding PeLysM genes by homologous recombination, to generate the ΔPeLysM1, ΔPeLysM2, ΔPeLysM3 and ΔPeLysM4 null mutants, respectively; (B) PCR amplification of the wild type, ΔPeLysM1, ΔPeLysM2, ΔPeLysM3 and ΔPeLysM4 null mutants and their respective ectopic mutants with diagnostic primers; (C) RT-PCR for ΔPeLysM1, ΔPeLysM2, ΔPeLysM4 or qRT-PCR for ΔPeLysM3 to compare the expression of LysM genes in wild type (P.e100), null (Δ) and ectopic (E) mutants in apples. 37S ribosomal protein s24 was used as a control. RGE–relative gene rxpression was calculated from Cq values using a ΔΔCq method [38]. Arrows in figures indicate the primers used. Arrows connected with line shows expected PCR product. Original gel images are available in S1 Appendix. (DOCX) [file pone.0186023.s007.docx]

S1
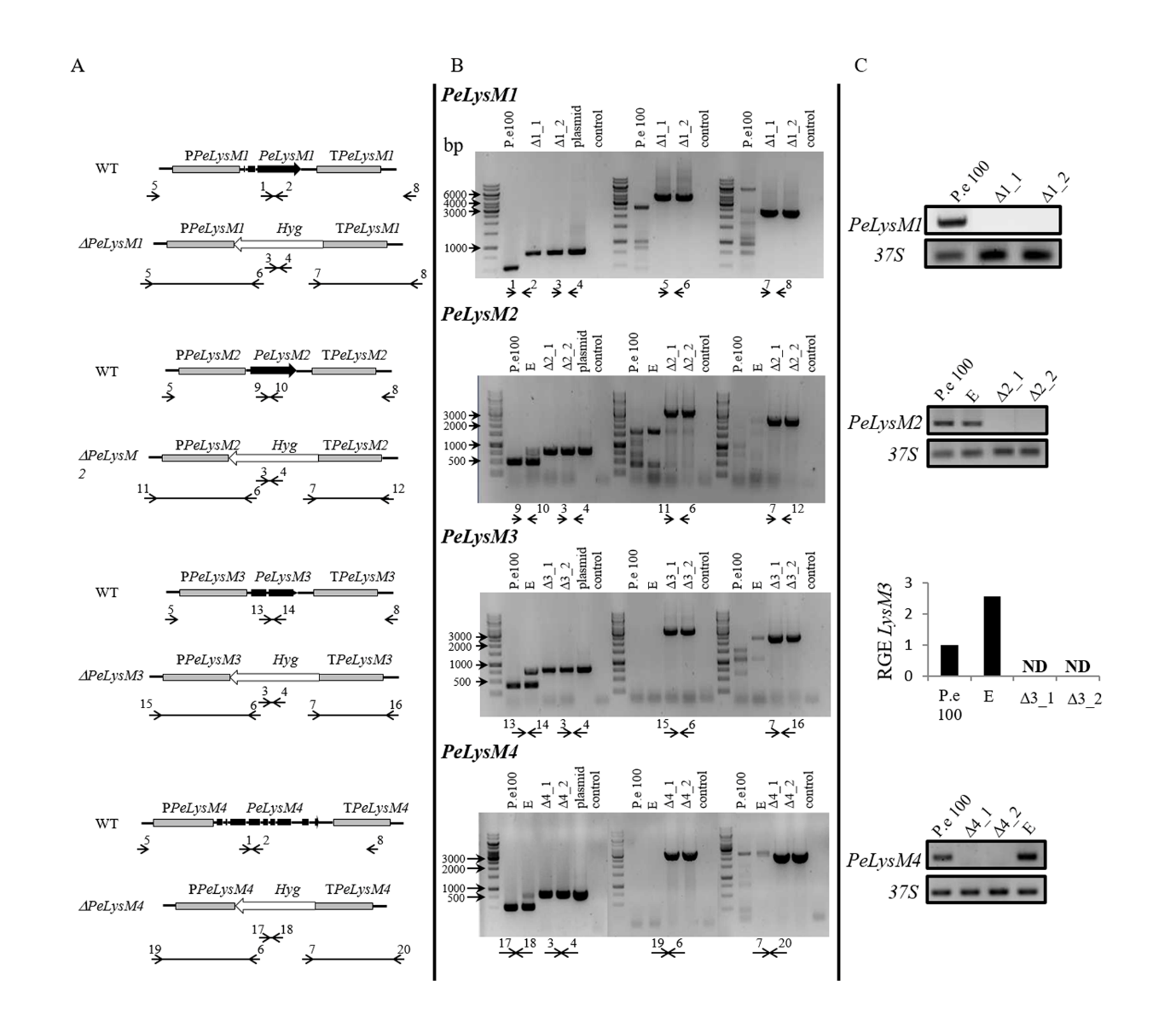
 Fig.: Deletion of PeLysM1, PeLysM2, PeLysM3 and PeLysM4 in Penicillium expansum PE100.

(A) Diagram of the wild type and deleted PeLysM1, PeLysM2, PeLysM3 and PeLysM4 loci. The hygromycin selectable marker present in the T-DNA of plasmids pRFHU2-PeLysM1, pRFHU2-PeLysM2, pRFHU2-PeLysM3 and pRFHU2-PeLysM4 replaces the corresponding PeLysM genes by homologous recombination, to generate the ΔPeLysM1, ΔPeLysM2, ΔPeLysM3 and ΔPeLysM4 null mutants, respectively; (B) PCR amplification of the wild type, ΔPeLysM1, ΔPeLysM2, ΔPeLysM3 and ΔPeLysM4 null mutants and their respective ectopic mutants with diagnostic primers; (C) RT-PCR for ΔPeLysM1, ΔPeLysM2, ΔPeLysM4 or qRT-PCR for ΔPeLysM3 to compare the expression of LysM genes in wild type (P.e100), null (Δ) and ectopic (E) mutants in apples. 37S ribosomal protein s24 was used as a control. RGE – relative gene rxpression was calculated from Cq values using a ΔΔCq method [38]. Arrows in figures indicate the primers used. Arrows connected with line shows expected PCR product. Original gel images are available in S1 appendix.
