# Supplementary material for: Identification and characterization of LysM effectors in Penicillium expansum
Source: PLoS One. 2017 Oct 30;12(10):e0186023. doi: 10.1371/journal.pone.0186023 (PMC5662087; doi:10.1371/journal.pone.0186023)
Supplement: S2 Fig — Average number of spore produced after 11 days of growth on potato dextrose agar medium based on three independent experiments. Bars indicate standard error. Letters indicate significant differences at P<0.05 based on nested one-way ANOVA followed by Tukey’s honest significant difference (HSD) test. (DOCX) [file pone.0186023.s008.docx]

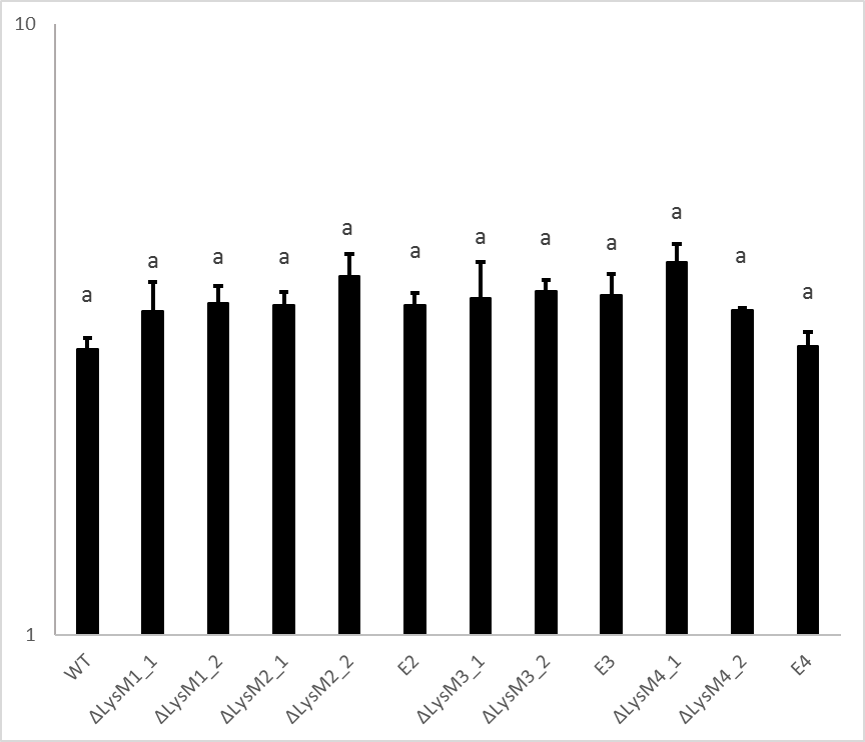


S2 Fig.: Effect of targeted deletion of *PeLysM1*, *PeLysM2*, *PeLysM3* and *PeLysM4* on spore production. Average number of conidia produced after 11 days of growth on potato dextrose agar medium based on three independent experiments. Bars indicate standard error. Letters indicate significant differences at *P*<0.05 based on nested one-way ANOVA followed by Tukey’s honest significant difference (HSD) test.
