# Supplementary material for: Identification and characterization of LysM effectors in Penicillium expansum
Source: PLoS One. 2017 Oct 30;12(10):e0186023. doi: 10.1371/journal.pone.0186023 (PMC5662087; doi:10.1371/journal.pone.0186023)
Supplement: S3 Fig — Five out 37 of positive clones are presented. The LysM domain (240 nt) from PeLysM1 was amplified by PCR, fused with the GAL4 DNA binding domain in the pGBKT7 vector and used as a bait. Empty vector was used as negative control. The prey library was constructed from total RNA isolated from ‘Royal Gala’ apple fruit collected at three different stages of development (early, mid-season, and mature). The library was packaged into the prey vector fused to the GAL4 activation domain. The candidate yeast cells were re-streaked several (3–4) times on SD/-Leu-Trp (DDO)/X selective medium. Each time a single blue colony, indicating a positive interaction (blue), was picked for re-streaking. All positive interactions (clones) were confirmed by patching on high stringency screening medium (SD/-Ade-His-Leu-Trp (QDO)/X/A). In order to further confirm that the interactions were genuine, prey plasmids from E. coli were transformed into the Y2H yeast strain containing the bait plasmid and grown on selective medium QDO/X/A. (DOCX) [file pone.0186023.s009.docx]

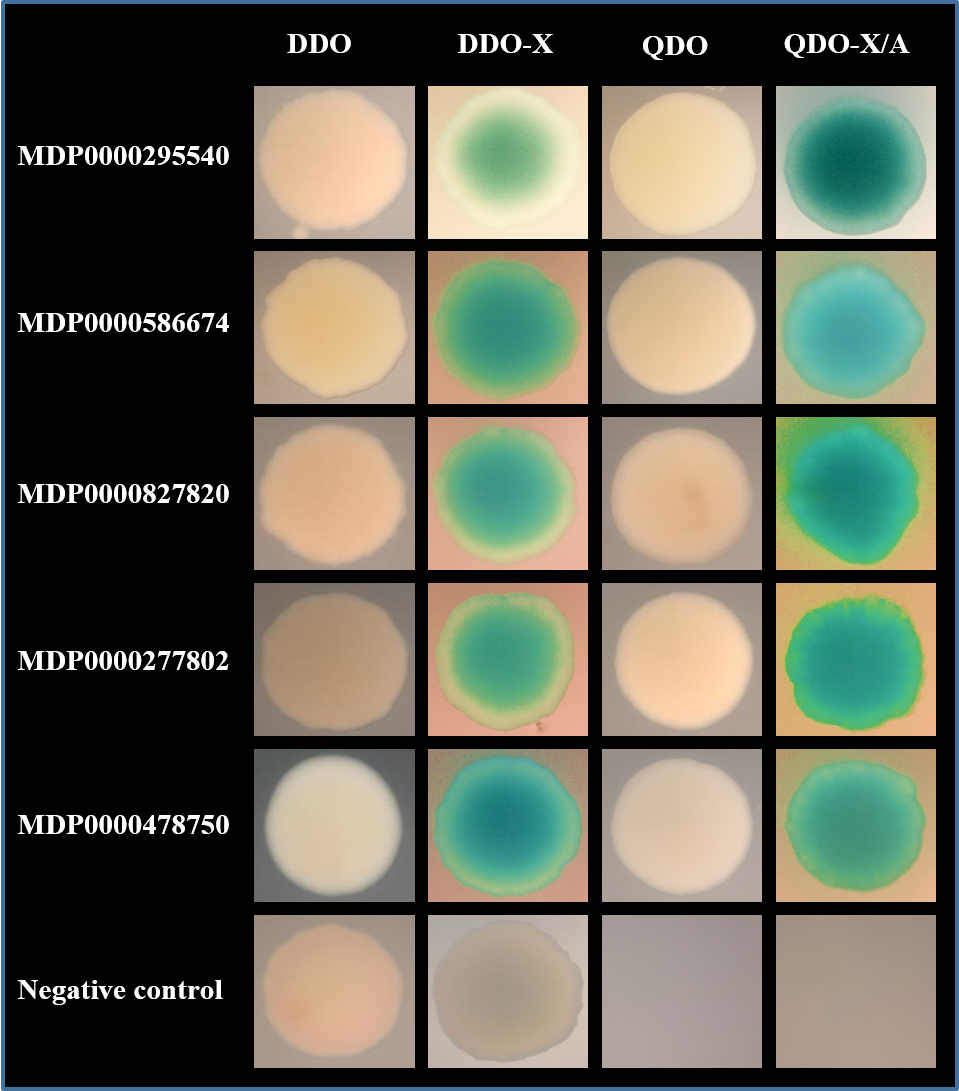


S3 Fig.: Partial results of the yeast-two hybrid analysis of the interactions between LysM from *P. expansum* and apple proteins. Five out 37 of positive clones are presented. The LysM domain (240 nt) from *PeLysM1* was amplified by PCR, fused with the GAL4 DNA binding domain in the pGBKT7 vector and used as a bait. Empty vector was used as negative control. The prey library was constructed from total RNA isolated from ‘Royal Gala’ apple fruit collected at three different stages of development (early, mid-season, and mature). The library was packaged into the prey vector fused to the GAL4 activation domain. The candidate yeast cells were re-streaked several (3-4) times on SD/-Leu-Trp (DDO)/X selective medium. Each time a single blue colony, indicating a positive interaction (blue), was picked for re-streaking. All positive interactions (clones) were confirmed by patching on high stringency screening medium (SD/-Ade-His-Leu-Trp (QDO)/X/A). In order to further confirm that the interactions were genuine, prey plasmids from *E. coli* were transformed into the Y2H yeast strain containing the bait plasmid and grown on selective medium QDO/X/A.
