# Supplementary material for: Identification and characterization of LysM effectors in Penicillium expansum
Source: PLoS One. 2017 Oct 30;12(10):e0186023. doi: 10.1371/journal.pone.0186023 (PMC5662087; doi:10.1371/journal.pone.0186023)
Supplement: S1 Appendix — (PPTX) [file pone.0186023.s010.pptx]

## Slide 1
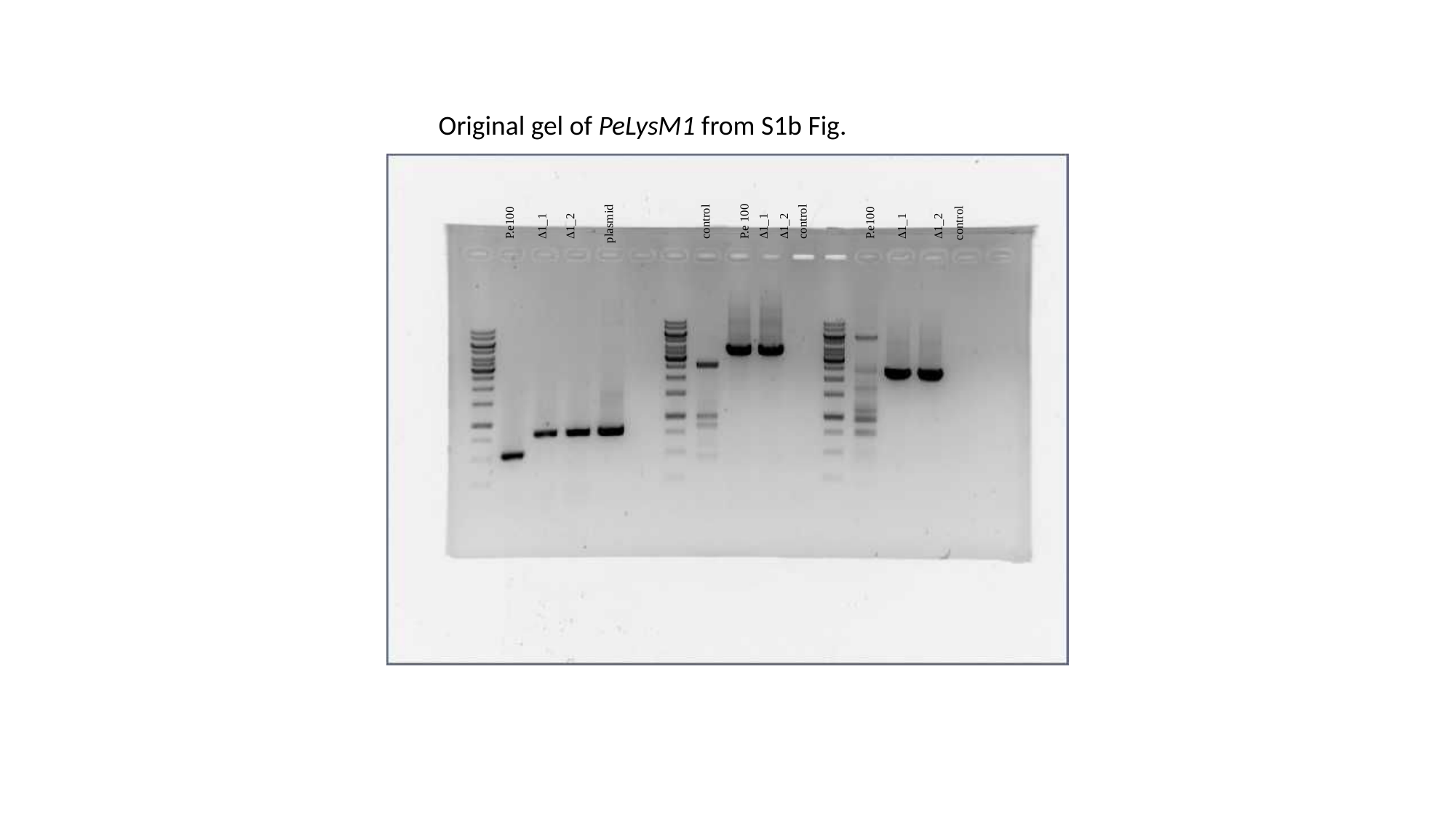

Original gel of PeLysM1 from S1b Fig.
plasmid
Δ1_2
Δ1_2
Δ1_1
Δ1_1
control
Δ1_1
Δ1_2
control
P.e100
P.e100
P.e 100
control

## Slide 2
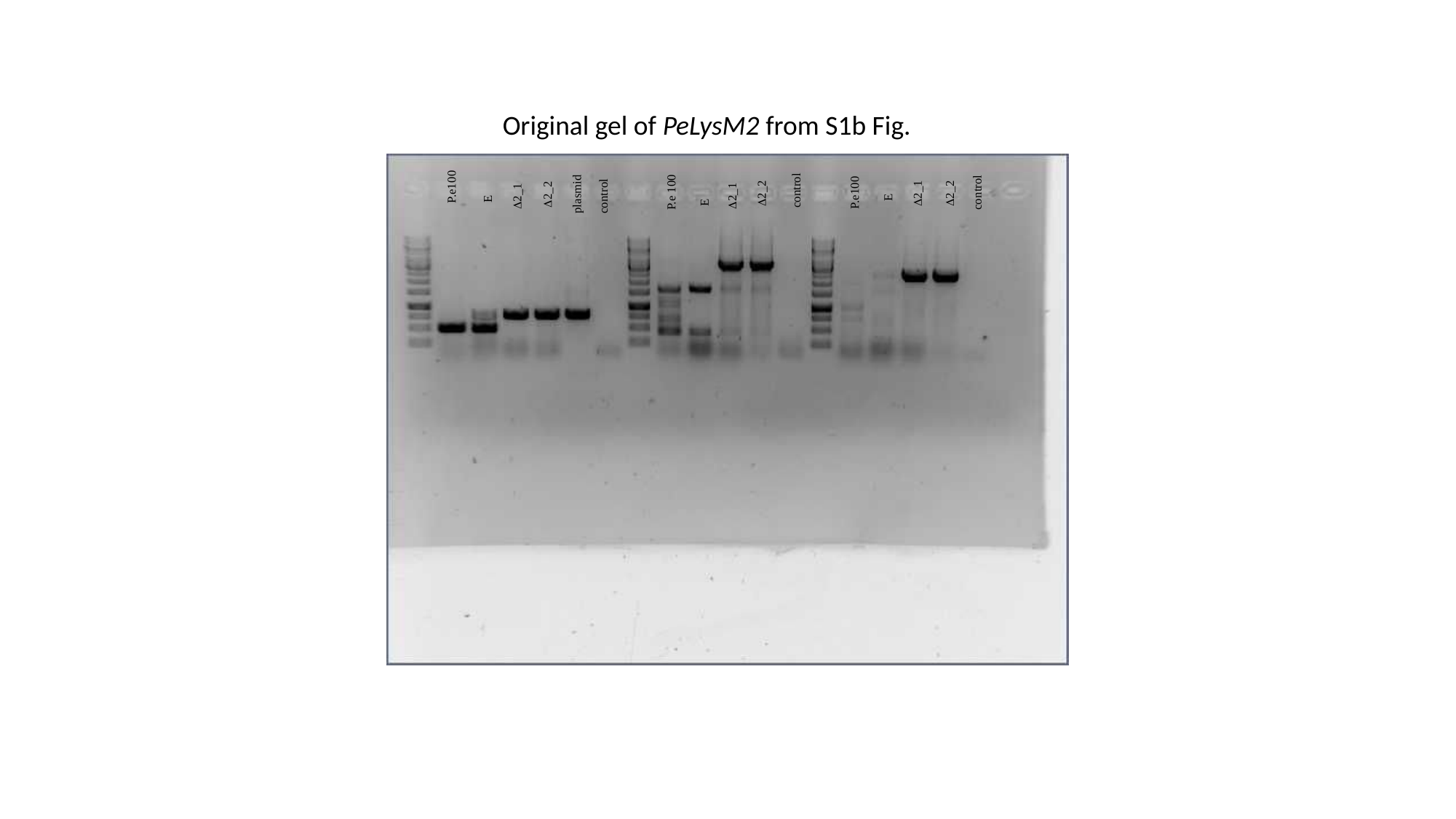

Original gel of PeLysM2 from S1b Fig.
E
P.e100
Δ2_2
Δ2_2
Δ2_1
control
P.e100
E
Δ2_1
Δ2_1
P.e 100
control
Δ2_2
plasmid
control
E

## Slide 3
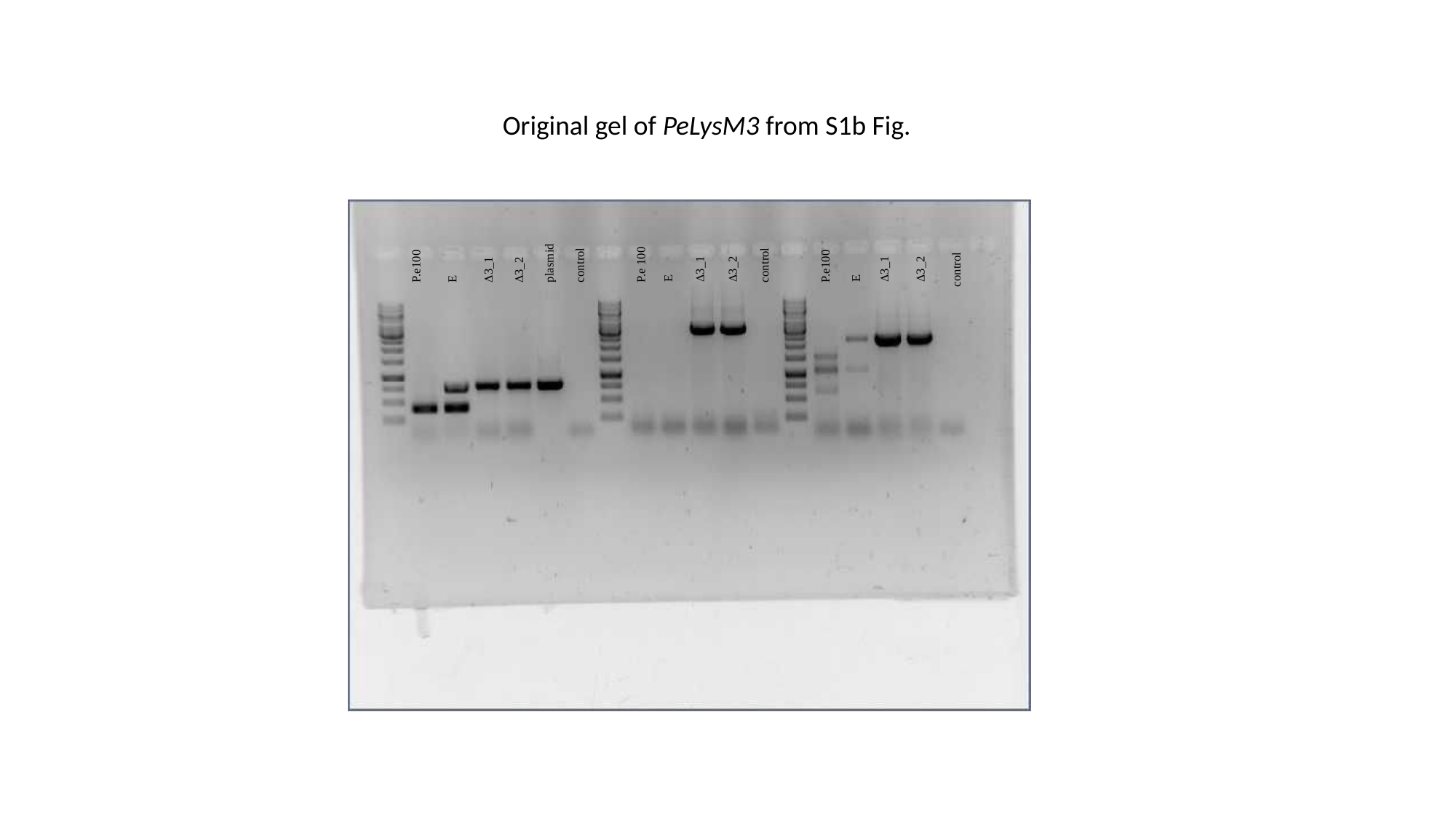

Original gel of PeLysM3 from S1b Fig.
Δ3_1
Δ3_1
Δ3_2
E
plasmid
P.e100
control
P.e 100
E
control
P.e100
Δ3_2
Δ3_2
control
Δ3_1
E

## Slide 4
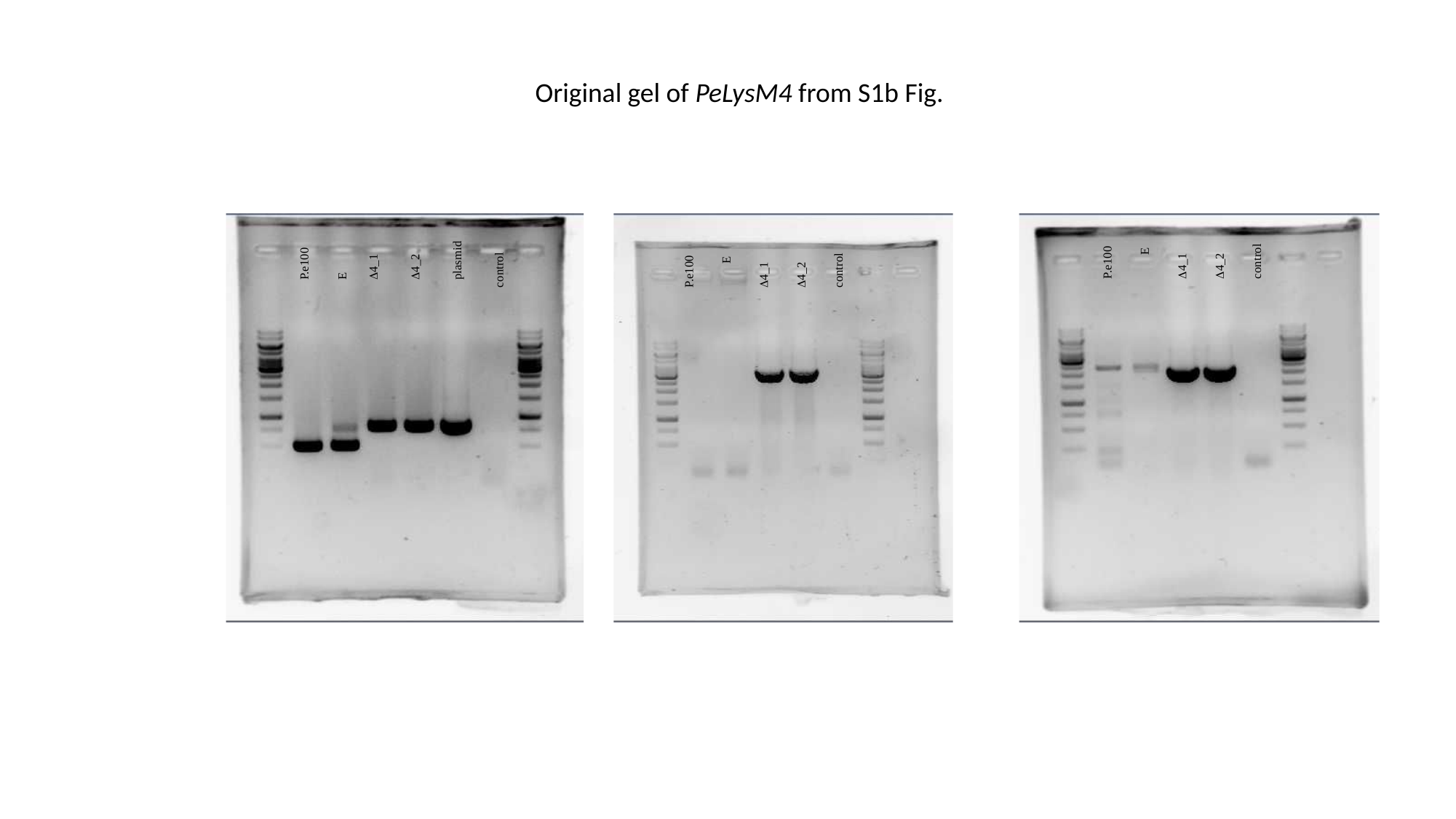

Original gel of PeLysM4 from S1b Fig.
E
E
P.e100
Δ4_1
Δ4_2
control
E
Δ4_1
plasmid
P.e100
Δ4_2
control
P.e100
Δ4_1
Δ4_2
control

## Slide 5
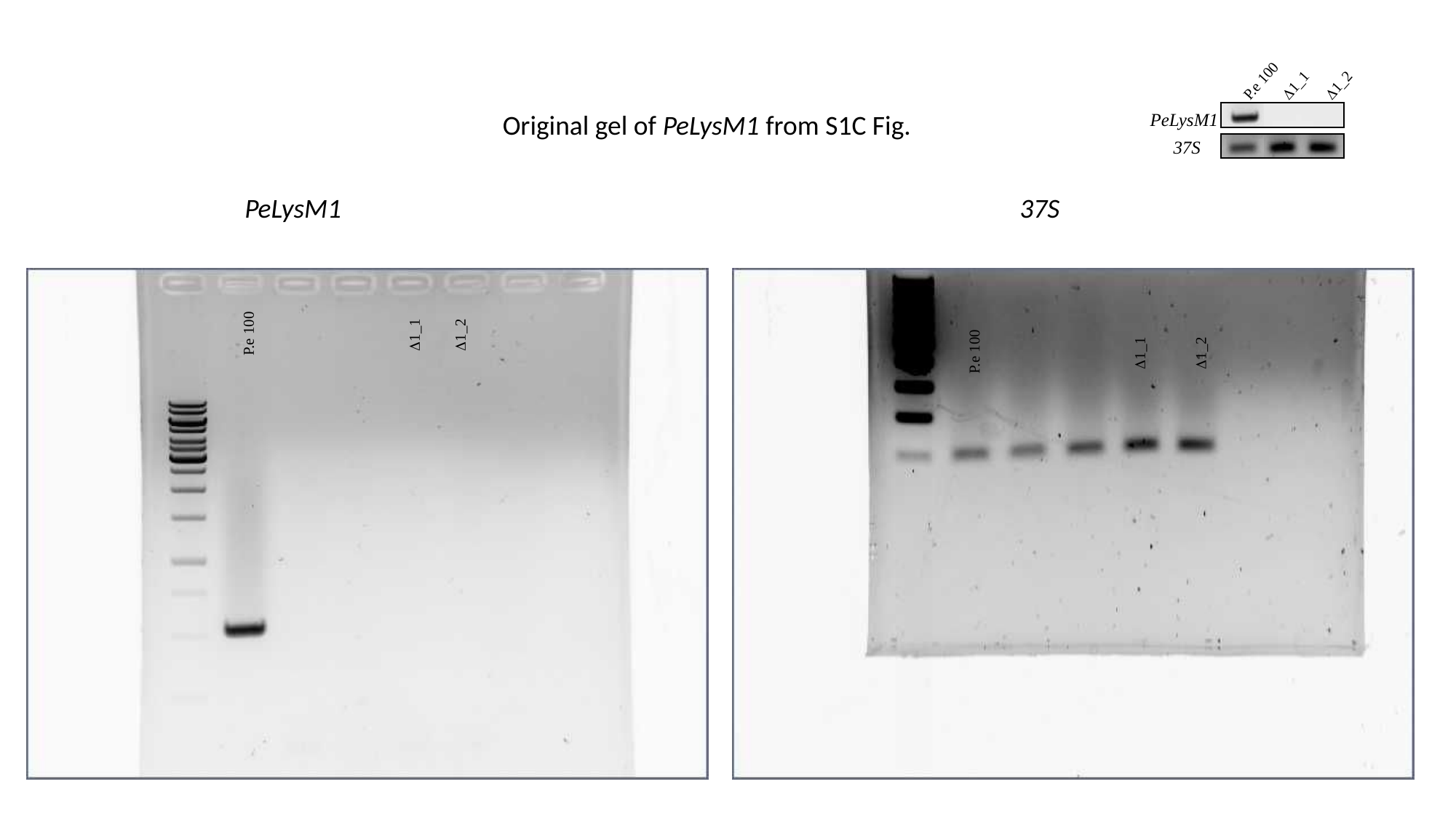

P.e 100
Δ1_1
Δ1_2
PeLysM1
37S
Original gel of PeLysM1 from S1C Fig.
PeLysM1
37S
Δ1_1
P.e 100
Δ1_2
Δ1_2
Δ1_1
P.e 100

## Slide 6
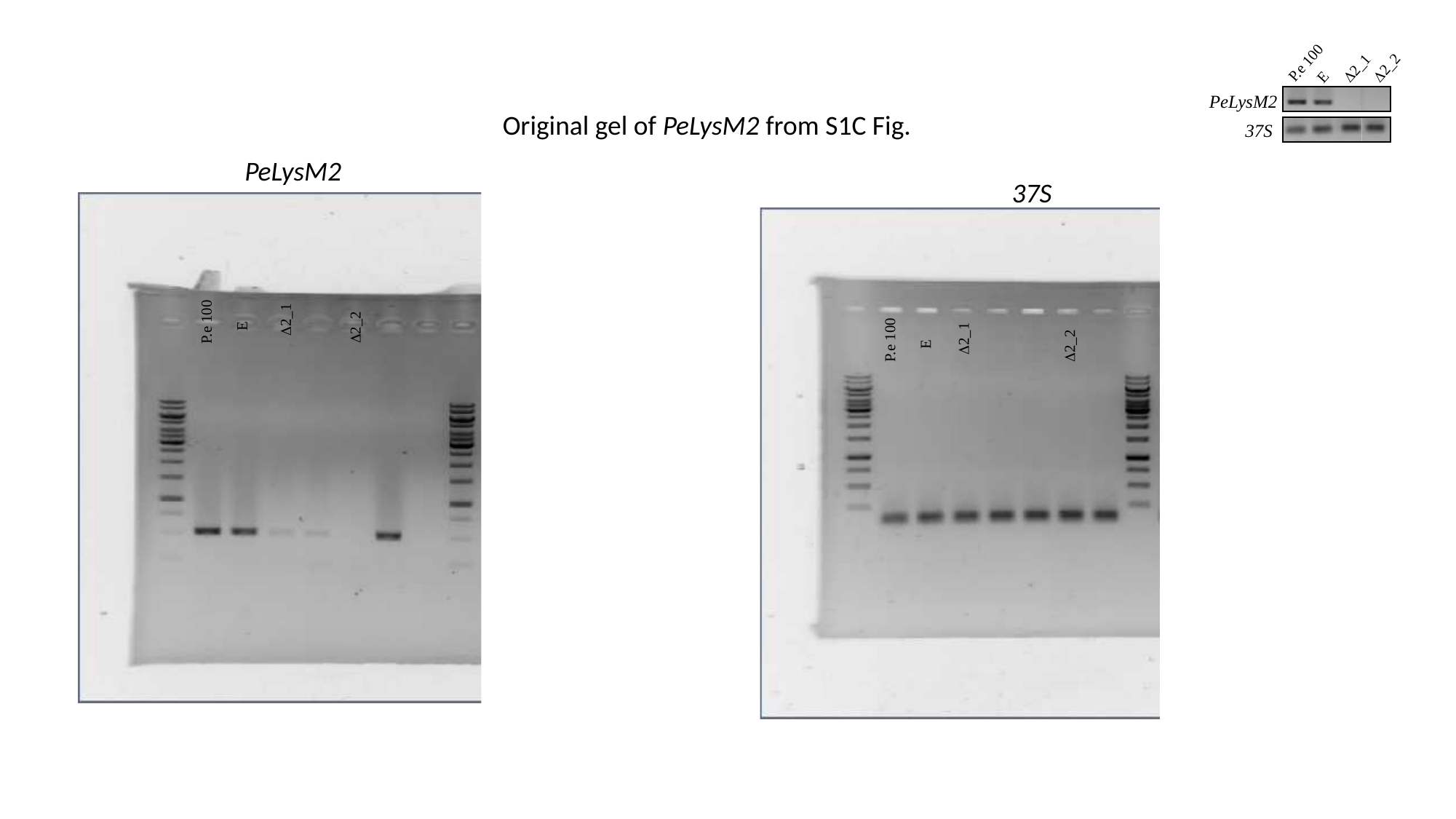

P.e 100
Δ2_2
Δ2_1
E
PeLysM2
Original gel of PeLysM2 from S1C Fig.
37S
PeLysM2
37S
E
P.e 100
Δ2_1
Δ2_2
E
P.e 100
Δ2_1
Δ2_2

## Slide 7
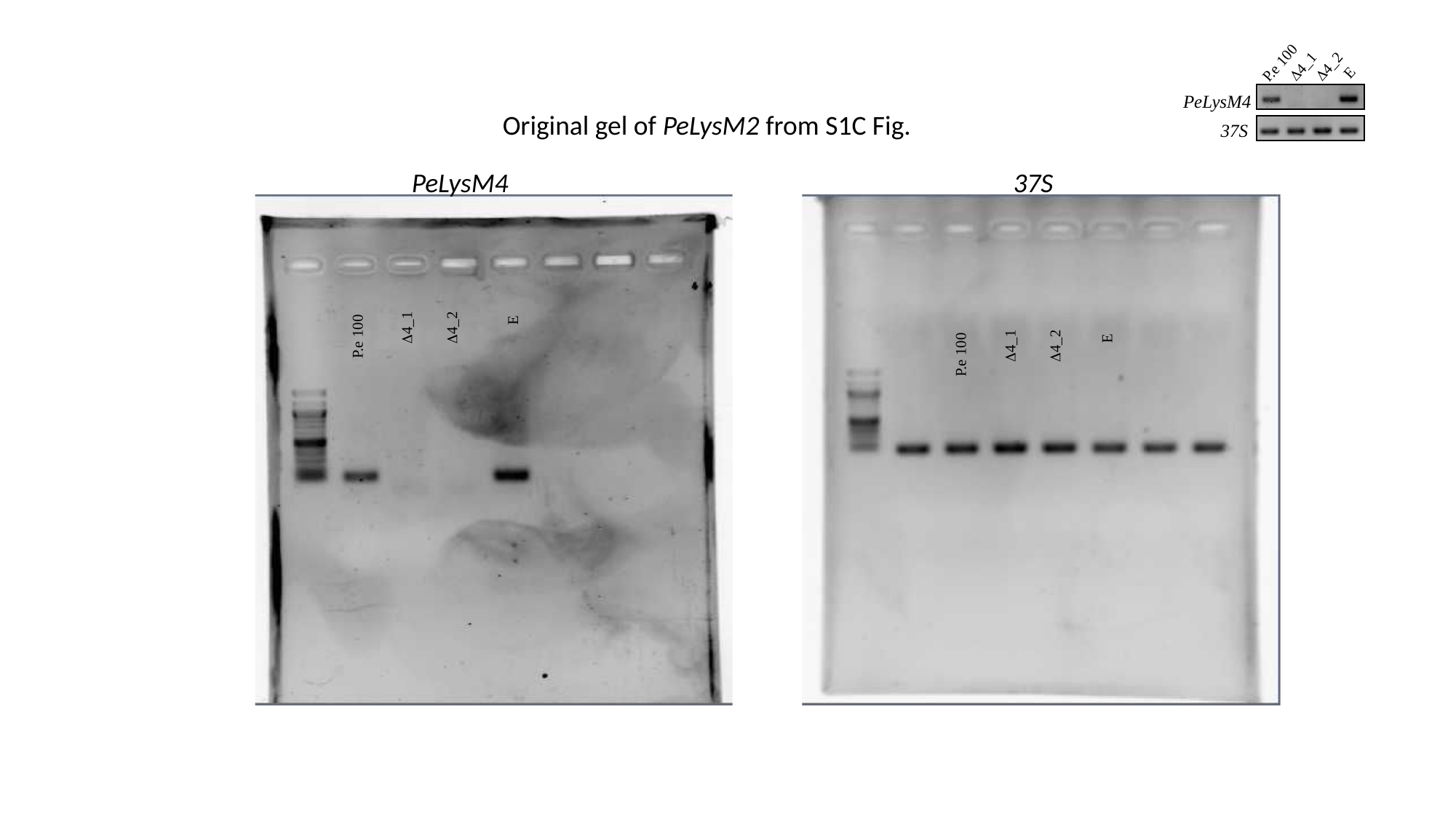

P.e 100
E
Δ4_2
Δ4_1
PeLysM4
Original gel of PeLysM2 from S1C Fig.
37S
PeLysM4
37S
E
E
Δ4_2
Δ4_1
P.e 100
Δ4_2
Δ4_1
P.e 100
